# Characterization of Hydrogel Deformation Using Two-Parameter Hyperelastic Models

**DOI:** 10.3390/gels12020171

**Published:** 2026-02-14

**Authors:** Joseph M. Scalet, Faiz Mandani, Stevin H. Gehrke

**Affiliations:** Department of Chemical and Petroleum Engineering, University of Kansas, Lawrence, KS 66045, USA; jmscalet@ku.edu (J.M.S.);

**Keywords:** hydrogels, polymer networks, rubberlike elasticity, hyperelastic models, Mooney–Rivlin, mechanical properties, modulus, PEGDA

## Abstract

Hyperelastic models for the deformation of hydrogels were evaluated as alternatives to the widely used neo-Hookean model. Poly(ethylene glycol diacrylate) (PEGDA) was synthesized via photopolymerization, with precursor molecular weights from 700 to 4000 Da and synthesis concentrations between 5 and 30 wt% in water. Hydrogels are often modeled as neo-Hookean solids; this model holds only over a limited strain range. To model deformation over a broader range and seek additional insight into gel network structures, the Mooney–Rivlin, Ogden, Rubinstein–Panyukov, and Localization models were applied to uniaxial compression data and their fits assessed against “Mooney plots” of reduced stress versus the inverse extension ratio. The Ogden model best fits the stress–strain curves to higher ratios and the reduced stress plots over the broadest range of formulations. The Localization and Rubinstein–Panyukov models fit well above c*, the overlap concentration, capturing low-strain behavior and the observed maxima under compression in Mooney plots. The Mooney–Rivlin model fit the stress–strain curves but was unable to fit the reduced stress plots. The Localization and Rubinstein–Panyukov model parameters suggest that entanglements play a significant role at all concentrations, with their contribution decreasing as the network concentration increases. This demonstrates the potential of using two-parameter models to understand the deformation of hydrogels.

## 1. Introduction

Hydrogels are key materials used in various medical applications, including drug delivery, tissue engineering, and wound dressings, as well as non-medical applications such as the creation of membranes and superabsorbents [1,2]. For tissue engineering, models that capture the full range of deformation under stress, including moduli and failure properties, are needed [3]. Other applications require them to withstand or apply specific forces or maintain precise swelling control [4], which are functions of network structure, often modeled in terms of the crosslink density [5]. Other network properties, such as mesh size and molecular weight between crosslinks, are used in models of solute diffusion in a gel, which is crucial for applications such as drug delivery systems and gel electrophoresis. Network properties, such as crosslink density and mesh size, are often obtained by mechanically deforming the hydrogel and measuring the amount of stress required for a given displacement. The neo-Hookean model of rubber elasticity is widely used to characterize the deformation behavior of hydrogels, and the modulus found from this model is used to calculate such network parameters [6]. However, the limitations of this model for characterizing the deformation behavior of gels have been noted [7,8,9]. While many other techniques for the evaluation of hydrogel molecular structures are used, such as small-angle neutron scattering (SANS), time-domain NMR, and solute permeation, mechanical characterization is the most common and straightforward to perform [10,11,12]. Thus, it is important to identify models that accurately model hydrogel deformation behavior, both because of the importance of the mechanical characteristics and also to recognize the limitations in the use of the neo-Hookean model when used to determine network parameters. The specific goal of this work is to demonstrate the potential of two-parameter models that are effectively extensions of the neo-Hookean models to better characterize deformation behavior. Poly(ethylene glycol diacrylate) (PEGDA) has been used as the model hydrogel because of its importance in biomedical and other applications, and because its mechanical properties and microstructure have been widely examined.

Two quite different approaches to modeling the deformation behavior of hydrogels have been used in the literature: those that treat the gel as a single-phase elastic or viscoelastic material, or those that treat the gel as a multiphase material comprising a polymer network phase and a solvent phase, and thus as a poroelastic or poroviscoelastic material [13]. In the former models, the gel is treated as a constant volume, incompressible solid. In the latter, the flow of the solvent through the pores upon deformation is considered as an integral part of the deformation behavior. The length and time scales are key to model selection. If the length scale and time scale were such that fluid flow under deformation occurred, the poroviscoelastic models can include those effects. For gels on the time scales evaluated in typical compression experiments of the type conducted in this work, constant-volume, incompressible behavior is observed. PEGDA has been modeled by both types of models, but, for experiments of the type in this paper, hyperelastic models have been applied [13,14,15,16].

While this work focuses exclusively on hyperelastic models, it is worth noting that this is not the only way to characterize PEGDA hydrogels. When characterizing the mechanical responses of hydrogels and similar materials, such as cartilage and biological tissues, there are many important factors to consider and multiple methods for quantifying these materials [17]. While there are many models and methods for characterizing hydrogels, broadly speaking, the two most common approaches are either to model the mechanical response of hydrogels as a multiphase material comprising a polymer network phase and a solvent phase using poroelastic models, or to treat them as single-phase elastic materials using viscoelastic models [13]. The key assumption of poroelastic models, which are based on mass transport, is the idea that the solvent phase is free to flow out of the material during compression, leading to a change in the Poisson ratio as the volume of the material is not conserved [14]. Viscoelastic models, which approach hydrogels from a thermodynamic approach, generally assume a negligible solvent flow and incompressibility over the experiment duration [18]. Both models can be valid for the same material, and, depending on the material and experiment, both might be needed to characterize the hydrogel [17]. One of the biggest considerations when determining if poroelastic effects need to be considered is the diffusion rate at which the solvent is expected to flow compared to the time scale of interest. In many experiments using unconfined uniaxial compression, the time scale over which poroelastic effects are expected to be observed is much longer than the experimental time scale, especially if the pore size of the gel is small, on the order of <10 nm [19]. In this paper’s case, the flow of the solvent from the hydrogel is expected to be negligible, and thus poroelastic effects can be ignored [16].

### 1.1. Hyperelastic Models

A hydrogel’s deformation response to uniaxial tension and compression can be described with one of many hyperelastic models—mathematical equations describing an elastic material’s stress–strain relationship [20,21]. These models are built from classical rubber elastic theory and assume that the deformation behavior is governed by the entropic effects of chain deformation, and that the enthalpic contributions are small enough to be ignored in the development of models that capture the key characteristics of the deformation behavior [22,23,24].

The neo-Hookean model is the most widely used hyperelastic model for hydrogels. The neo-Hookean equation is most commonly written as follows [25,26]:

(1) $$\sigma =G(\lambda -\lambda^{-2})$$

where σ represents the engineering stress, and λ represents the deformation ratio and is defined as λ $=L/L_{0}$, where L is the deformed length, L_o_ is the undeformed length, and G is the shear modulus. This equation and the advanced models provided in the following section are often presented in terms of the reduced stress [f*], as given in Equation (2) [27,28]:

(2) $$[f^{*}]=\frac{\sigma }{\left(\lambda -\lambda^{-2}\right)}$$

The neo-Hookean model can be applied to both tension and compression in elastomers and can also be written in terms of the Young’s or compressive modulus E, since E ≈ 3 G for elastomers due to their incompressibility (leading to a Poisson ratio ≈ 0.5) [29]. In the literature, hyperelastic models are generally presented in terms of G rather than E. Using moduli found from the neo-Hookean model, other useful parameters to characterize gels are commonly calculated using appropriate theories, such as the average mesh size or crosslink density [6]. However, the neo-Hookean model is derived under several assumptions, including that the chains have a Gaussian distribution of chain extensions at all deformations and that the material is homogeneous [21]. However, the neo-Hookean model generally deviates from the observed stress–strain behavior at higher strains. Thus, numerous alternative models for elastomer deformation have been developed over the decades [21,30,31].

As water-swollen elastomers, hydrogels are generally expected to be ideal neo-Hookean materials. The dilution of the chains is expected to limit their interactions, which lead to strain-dependent effects [21]. Thus, the neo-Hookean model is routinely used to characterize hydrogels, enabling the determination of G and calculation of other structural parameters from this measurement. This assumption is valid for many hydrogels with modest strains on the order of 20%, with deviation steadily increasing as strain increases [4,10]. However, for some hydrogels with complex structures, the neo-Hookean model fits the data very poorly [32,33,34,35]. Nonetheless, alternative models are rarely considered [33]. Although many other models have been developed for solid elastomers, they have been applied to hydrogels only rarely. There have been examples where the Mooney–Rivlin model has been used; however, the parameter C_2_, which characterizes the strain dependence, has generally been reported as negligible [36,37].

Recently, several papers have identified the limitations of the neo-Hookean model in accurately modeling the deformation behavior of hydrogels, particularly under compression to meaningful strains, and have proposed two-parameter hyperelastic models as an alternative. Having noted that many hydrogels exhibit non-linear stress–strain responses, Nedrelow et al. utilized the Ogden model for the analysis of pentenoate-modified hyaluronic acid (PHA) and PHA-PEGDA hydrogels because of its ability to capture non-linear behavior. Their findings showed that the Ogden model can accurately fit the stress–strain curve for a variety of hydrogels over the full range of compressive strain up to failure at approximately 50%, which the neo-Hookean model could not achieve [7]. Therefore, they recommended the Ogden model for general use in the development of hydrogels for tissue engineering and other biomedical applications of hydrogels. Tang et al. also demonstrated that the neo-Hookean model was insufficient to adequately interpret the trends in the stress–strain behavior of the important synthetic hydrogel PEGDA as a function of the formulation. However, they were able to use the two-parameter Yeoh model to fit the stress–strain behavior in terms of parameters related to synthesis variables [9]. Thus, these recent publications highlight the need to investigate a range of hyperelastic models that can more accurately capture the deformation response of hydrogels and, ideally, provide insight into the hydrogel network.

### 1.2. Two-Parameter Hyperelastic Models

Numerous efforts and theories have been made to improve on the neo-Hookean model. Nearly all of these were models developed for solid elastomers, often using polydimethylsiloxane (PDMS) as the model polymer. The adoption of these for use in hydrogels has been limited. In this section, the qualitative categorization of the models is presented, along with the rationale behind the selection of four models for further analysis. The mathematical forms of the equations are then presented.

Broadly, hyperelastic models can be categorized into two types: phenomenological models, which aim to describe observed behavior in terms of empirical parameters, and mechanistic models, which are developed from theoretical models of polymer chain interactions under stress and seek to explain the underlying reasons for the observed behavior in terms of physical parameters. Although phenomenological models have the potential to fit deformation data better, their fitting parameters generally do not provide additional information about the nature of the material, unlike mechanistic models, whose parameters are related to the physical characteristics of the polymers [30,31,38]. However, mechanistic models are expected to be accurate only to the extent that the network aligns with the model’s assumptions.

#### 1.2.1. Phenomenological Models Examined

The most widely used phenomenological model is the Mooney–Rivlin model, which was developed in the 1940s [39]. Other phenomenological models have also been developed, generally sharing common features and converging on similar functional forms. For instance, the Yeoh model has been successfully correlated with PEGDA formulation variables by Tang et al. [9]. The Ogden model was developed specifically for soft biological tissues, has a somewhat different functional form from the models, and is recommended for general use in the development of hydrogels for tissue engineering. Thus, the Mooney–Rivlin and Ogden models were chosen for evaluation in this paper [30]. The Mooney–Rivlin model is derived as a Taylor series expansion of the work density function, though it is usually truncated after the first two terms [14,40]. Its functional form is given in Equation (3), and includes both strain-independent and strain-dependent terms [41,42]:

(3) $$\left[f^{*}]=2(C_{1}+\frac{C_{2}}{\lambda }\right)$$

where [f*] is the reduced stress (Equation (2)), λ is the deformation ratio as defined earlier and C_1_ and C_2_ are empirical parameters arising from the series expansion [29].

The Ogden model is also a two-parameter phenomenological model but has a different functional form from Mooney–Rivlin. As a result, it is not easily represented in terms of [f*] as the other models are, so it is presented in terms of the stress and deformation ratio. It adds a second empirical parameter a in the exponent of the deformation ratio, as shown in Equation (4), and can be related to the shear modulus, as given in Equation (5).

(4) $$\sigma =\frac{2G}{\alpha }(\lambda^{\alpha }-\lambda^{\left(\frac{-\alpha }{2}\right)})$$

where α is a nonlinearity parameter, and G is the shear modulus [27].

#### 1.2.2. Mechanistic Models Examined

The two mechanistic models chosen for evaluation are the Localization model and the Non-Affine Slip Tube Model (hereafter referred to by the authors’ names as the “Rubinstein–Panyukov model”), which have garnered notable interest for their application to gels [22,38,43]. Both have a basis in the Edwards tube model, which assumes that the chains are confined to a tube-like volume [30,31,38]. However, the models are derived quite differently. In the Rubinstein–Panyukov model, the two major deviations from the Edwards model are: (1) the Rubinstein–Panyukov model assumes virtual chains are randomly distributed in a Gaussian distribution of chain lengths in the network at preparation conditions, rather than attached to an elastic virtual background by uniformly distributed virtual crosslinks, and (2) in the volume the chains can deform non-affinely. The Localization model also employs the concept of chains being confined or “localized” to a tube-like region and focuses on how confined chains reduce the entropy of the network chains compared to unconstrained chains. It is described as a “minimal model” designed to be capable of capturing the key elements of the deformation behavior in a simple form. Both of these mechanistic models seek to understand the role entanglements play in the polymer network stress–strain response.

Both the Localization and the Rubinstein–Panyukov models [38,44] are two-parameter models. One of their key advantages is that they separate the contributions to the shear modulus into entanglement and network components. Thus, the deformation response to stress is separated into a contribution from permanent crosslinks (G_c_) and a contribution from entanglements (G_e_). In principle, G_c_ can be found from the theoretical crosslink density based on stoichiometry [45]. However, in practice, this can be difficult for gels such as PEGDA, which have complex structures [14,46,47]. Instead, it can be estimated from small-strain behavior, over which it is assumed that neo-Hookean behavior is valid [9].

The Rubinstein–Panyukov model is given in Equation (6), written in terms of the reduced stress:

(5) [f*]=Gc+Ge0.74λ+0.61λ−0.5−0.35

where G_c_ and G_e_ are the covalent and entanglement contributions to the shear modulus, respectively. The denominator of the second term in this function was developed by the authors by curve fitting theoretical predictions to an invariant strain function.

The Localization model was developed by Gaylord and Douglas [38]. Both the Localization and Rubinstein–Panyukov models aim to achieve similar goals, but the differences in their functional forms result in different fits and parameter values. The Localization model uses the same nomenclature as the Rubinstein–Panyukov model and is given in Equation (7):

(6) [f*]=Gc+Ge1−λ(−32)(λ−λ−2)

### 1.3. PEGDA as a Model Hydrogel

In this work, we use poly(ethylene glycol diacrylate) (PEGDA) as the basis for evaluating these models. Due to their favorable cytocompatibility, swelling properties, and ease of modification, PEGDA hydrogels have been widely used in biomedical and other applications. Recent work on PEGDA has focused on utilizing PEGDA as a functional material for biomedical applications such as 3D printing or drug delivery, which makes it an important material to understand thoroughly [48,49,50,51]. Upon the synthesis of PEGDA, polyacrylate kinetic chains are formed and connected by PEG linking chains. Because of PEGDA’s utility and complex microstructure, there is an extensive body of literature on its microstructure and properties that it helps to consider when assessing the performance of two-parameter hyperelastic models [52,53].

Previous work has generally fitted the compression data of PEGDA hydrogels to the neo-Hookean model over a limited strain range, often only up to ~20%, including our previously published work, where we applied the neo-Hookean model to PEGDA and related gels. We noted consistent limitations in the performance of the neo-Hookean model in prior publications, but this is our first effort to go beyond the Mooney–Rivlin model [32,54]. Other authors have also demonstrated the need for alternative models that can more effectively capture the full range of PEGDA hydrogel stress–strain behavior [8,55]. Therefore, in this work, PEGDA is used as a hydrogel model to examine the performance of two-parameter hyperelastic models in correlating compression data. Additionally, for the mechanistic models, the potential value of these models in providing insight into the origins of deviations from the neo-Hookean model is assessed.

## 2. Results and Discussion

### 2.1. Swelling and Uniaxial Compression Measurements

**Figure 1:**
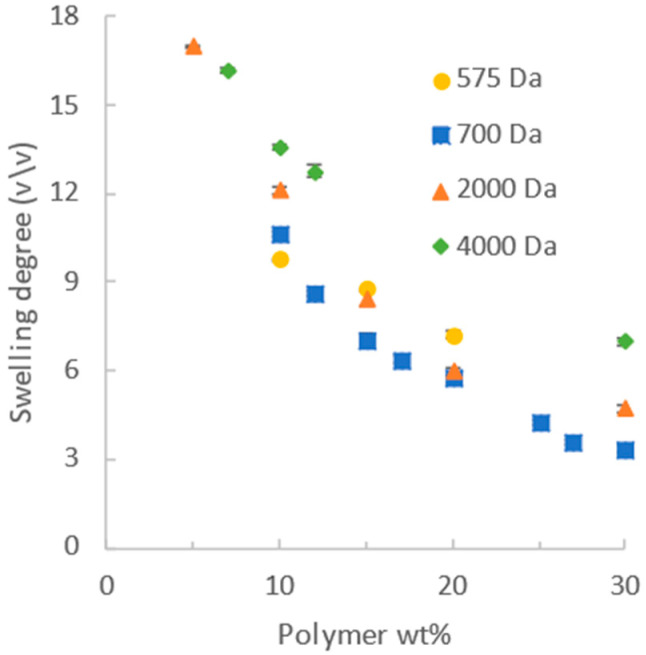
Swelling degree decreases as synthesis concentration increases for each PEGDA molecular weight.

As shown in Figure 1, increasing the concentration of the polymer precursor solution reduced the degree of swelling for all precursor molecular weights tested, from 575 to 4000 Da. When the concentration increased from 10% to 30%, the swelling of the PEGDA 2000 DA decreased from 15 to 4. Similar trends were observed for all molecular weights of PEGDA, with the overall magnitude of swelling decreasing modestly as the molecular weight decreased.

These trends are consistent with the prior literature and theoretical expectations [26,56]. The swelling of non-ionic crosslinked hydrogels is governed by their Flory–Huggins solubility parameters, crosslink densities, and precursor solution concentrations. Consistent with Flory–Rehner theory, the swelling degree of PEGDA hydrogels decreases with an increasing crosslink density and polymer precursor solution concentration [56].

**Figure 2:**
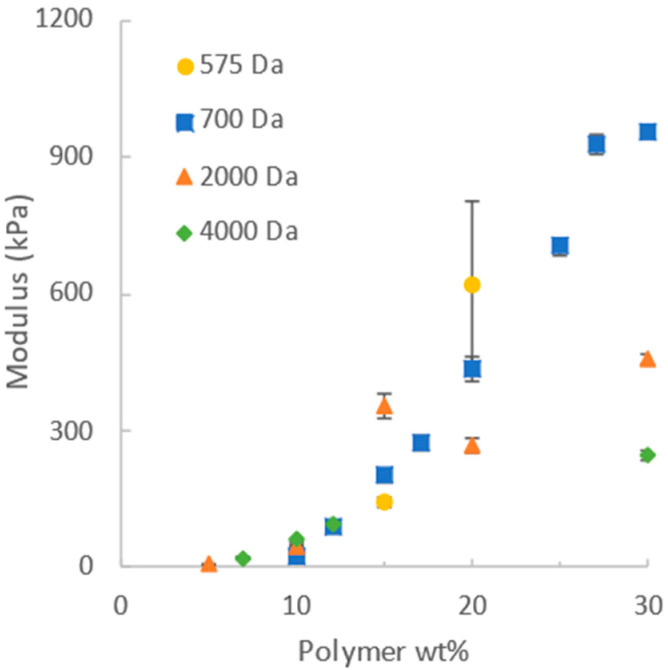
Shear modulus G, calculated using the neo-Hookean model, increases with increasing synthesis concentration for each molecular weight.

The stress–strain data obtained from uniaxial compression for the various molecular weights and concentrations were fitted to the neo-Hookean model conventionally using Equation (1) (strains up to 10%) to obtain values of the shear modulus G for the different formulations. These results are given in Figure 2, showing that the shear modulus increased over 50 times as the mass fraction of PEGDA at synthesis increased from 5% to 30% for 2000 Da PEGDA, with similar trends for the other molecular weights of PEGDA. These trends are consistent with the prior literature. However, the neo-Hookean model seldom fits the entire stress–strain curve data obtained for a hydrogel, so decisions must be made about which portion to use. In the literature, the range of data obtained for hydrogels and used to calculate the neo-Hookean G uses a linear portion of a plot of stress vs. λ − 1/λ^2^) as suggested by Equation (1), and may ignore very low and higher strains where significant deviations from linearity occur. Differences in judgment about which section of the data to use, as well as the variability in the PEGDA reagents and other experimental issues as identified by Tang et al., have led to a variability in previously reported moduli for PEGDA [9]. The use of two-parameter models can alleviate this problem.

### 2.2. Hyperelastic Modeling

**Figure 3:**
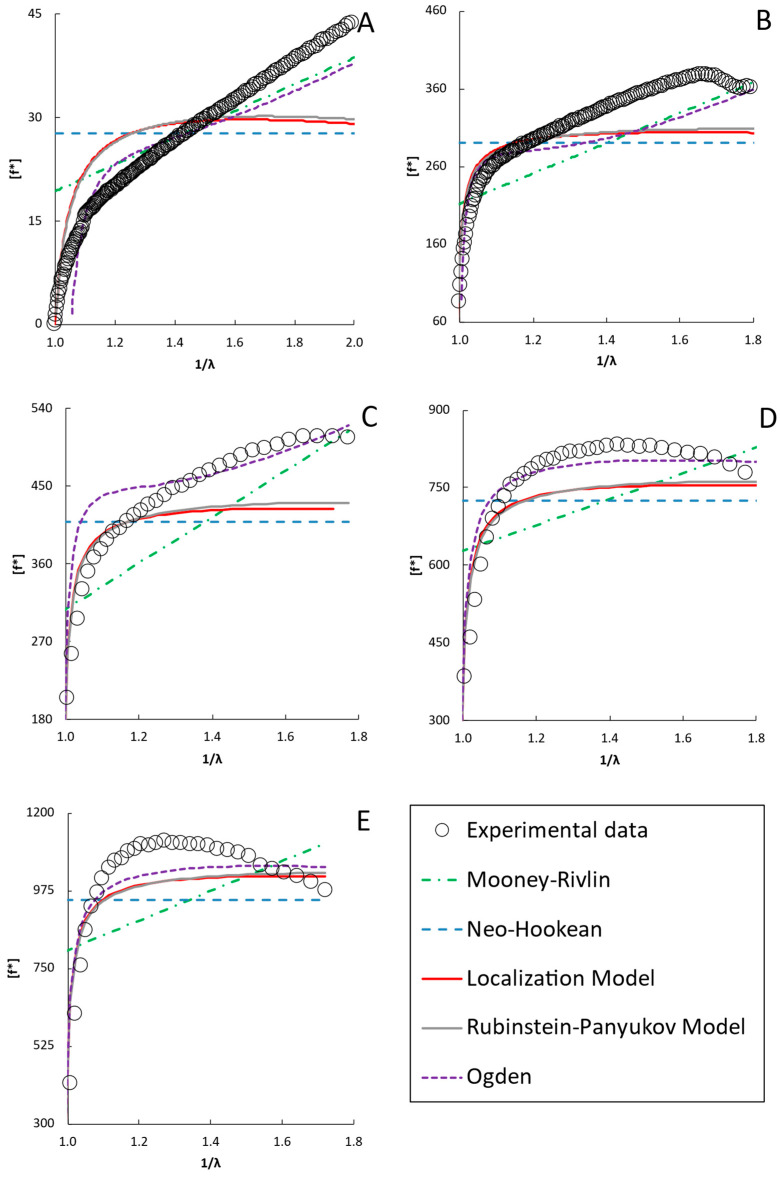
Plots of the various models fitted to a reduced-stress plot of PEGDA 700. Inset shows the same fittings displayed on a stress–strain plot. (**A**) 10 wt%, (**B**) 17 wt%, (**C**) 20 wt%, (**D**) 25 wt%, (**E**) 30 wt%. Enlarged versions of these figures along with plots of the stress vs. strain data from which these figures were prepared is provided in the Supplementary Information.

**Figure 4:**
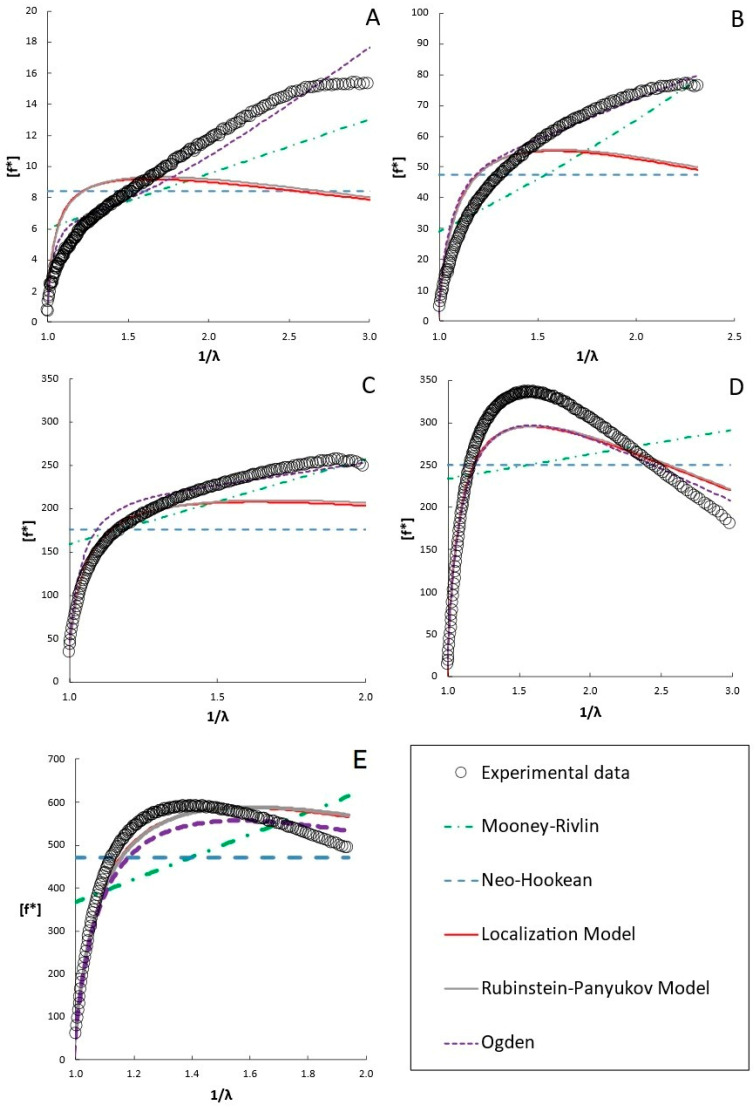
Plots of the various models fitted on a reduced-stress plot of PEGDA 2000 Da. Inset shows the same fittings displayed on a stress–strain plot. (**A**) 5 wt%, (**B**) 10 wt%, (**C**) 15 wt%, (**D**) 20 wt%, (**E**) 30 wt%. Enlarged versions of these figures along with plots of the stress vs. strain data from which these figures were prepared is provided in the Supplementary Information.

**Figure 5:**
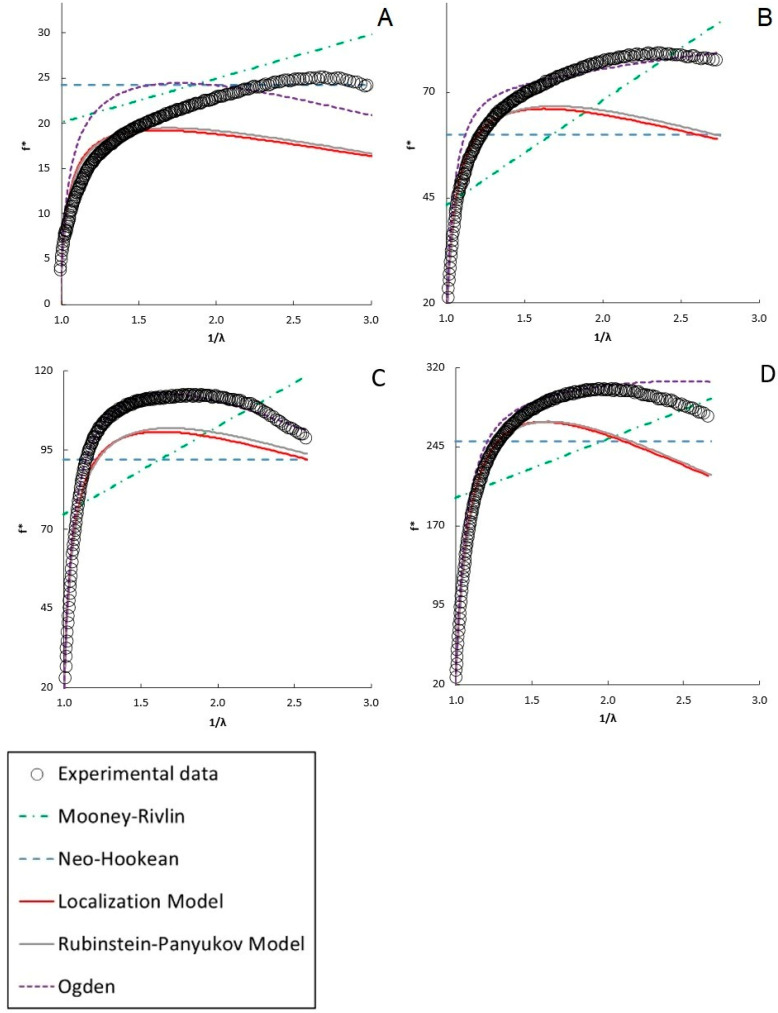
Plots of the various models fitted to a reduced-stress plot of PEGDA (4000 Da). Inset shows the same fittings displayed on a stress–strain plot. (**A**) 7 wt%, (**B**) 10 wt%, (**C**) 12 wt%, (**D**) 30 wt%. Enlarged versions of these figures along with plots of the stress vs. strain data from which these figures were prepared is provided in the Supplementary Information.

**Figure 6:**
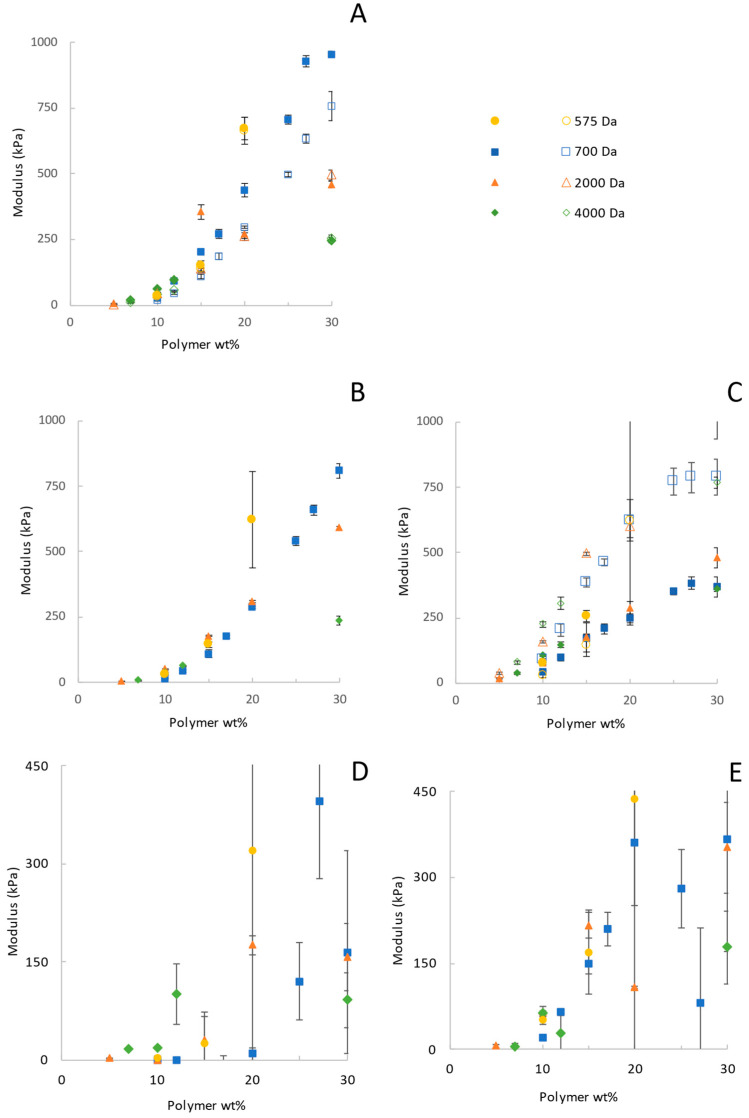
Fitted parameters for PEGDA of various molecular weights. (**A**) Shear modulus of the Ogden (open) and the neo-Hookean (closed) models (when fit to the entire strain range). (**B**) G_c_ (―) values used for the Rubinstein–Panyukov and Localization models. (**C**) Ge for the Rubinstein–Panyukov (closed) and Localization models (open). (**D**) C_1_ for the Mooney–Rivlin model and (**E**) C_2_ for the Mooney–Rivlin model. Note the large error in the Mooney–Rivlin parameters, which is indicative of the poor fitting of the model to the data.

As discussed in the introduction, numerous hyperelastic models have been proposed to analyze stress–strain behavior. Typically, these models can be fit to the stress–strain data or a plot of the reduced stress vs. 1/λ. Although fitting the data directly to the stress–strain plot is possible, many of the hyperelastic models yield similar R^2^ values, and thus such a fitting method does not discriminate well among models. That is, the models may deviate from the data in different ways that may yield similar statistics on fit quality. Instead, fitting the models to a plot of [f*] vs. 1/λ (a “Mooney plot”) better discriminates among the models, as discussed in Section 4.2.4 [30]. The model fits to the data are presented for selected molecular weights and concentrations in Figure 3, Figure 4, Figure 5 and Figure 6. A full presentation of the stress–strain and Mooney plots for all molecular weights and concentrations can be found in the Supplementary Materials. The parameters of the different models are presented in Figure 6 for all the molecular weights and concentrations examined.

Generally, each of the two-parameter models fits the stress–strain data well using the parameters obtained from the Mooney plots. They capture the general shape of the uniaxial compression data and the data itself quite well. In contrast, the neo-Hookean model generally underpredicts higher-strain data, as seen in the insets of Figure 3. However, the Mooney plots show that each model accounts for the deviations from neo-Hookean behavior differently and that not all models fit equally well for all formulations.

On the Mooney plots shown in Figure 3, Figure 4 and Figure 5 for PEGDAs −700 Da, 2000 Da, and 4000 Da, the data trends shift as the PEGDA concentrations at synthesis increase. The trends are similar for all molecular weights, so the analogous plots for the other molecular weights are given in the Supplementary Materials. At lower concentrations, the reduced stress increases monotonically, with the local slopes at higher inverse deformation ratios gradually declining until maxima are observed at higher PEGDA concentrations, above the overlap concentration c* for the precursor PEGDA. Neither the neo-Hookean nor the Mooney–Rivlin models can fit the data well at any concentration, neither in the initial deformation region nor at the maxima. This results from the fact that the neo-Hookean model has no strain dependence and the Mooney–Rivlin model predicts only a linear trend with an inverse deformation ratio, so neither can capture the curvature observed in the reduced stress (though, if the values of C_1_ and C_2_ are allowed to become negative, inconsistent with the underlying model, good empirical fits can be obtained with a global fit of the data using software like the structural mechanics module in COMSOL6.4). This highlights the shortcomings of these two models and the limitations of fitting data directly to the stress–strain curve, underscoring the need for better models to model the deformation behavior of hydrogels accurately.

Of the two-parameter models evaluated in the Mooney plots, the Ogden model fits the broadest range of data sets well, with the highest R^2^ values (R^2^ = 0.96–0.99 across all molecular weights and concentrations of PEGDA) among all the tested models across the entire strain range. The Rubinstein–Panyukov and Localization models fit the data similarly for all formulations, and fit the data nearly as well as the Ogden model at 20 and 30 wt%, above c* (12 wt% for PEGDA 2000) [56]. However, these models did not fit the data as well as the Ogden model below c*. These models fit the low-strain data (inverse deformation ratio approaching one) similarly to Ogden; however, they underpredict the data significantly beyond the low-strain range. However, even the Ogden model predicted a less steep decline beyond the maxima seen in the data.

Similar trends to those described for PEGDA 2000 were observed for PEGDA with molecular weights of 700 Da and 4000 Da, shown in Figure 3 and Figure 5 (additionally, PEGDA 575 and expanded figures for all the molecular weights are shown in the Supplementary Materials), with the Ogden model providing the most accurate representation of the data shape and best overall fit (R^2^ value over 0.98 for all samples) for the broadest range of data. Similarly to the observations with PEGDA 2000, the Localization and Rubinstein–Panyukov models did not accurately capture the curvature of the data at concentrations below c*, with the 4000 Da PEGDA showing a slightly higher average R^2^ for both models compared to the 2000 Da PEGDA. Given that the Rubinstein–Panyukov and Localization models are both based on the idea that the polymer chains are confined to a tube-like volume by their neighboring chains, it makes sense that they fail to capture the behavior of PEGDA below c*, the overlap concentration, where chain interactions are limited. The overlap concentrations for PEGDA 575, 700, 2000, and 4000 are 20 wt%, 18 wt%, 12 wt%, and 9 wt%, respectively [56].

Finally, the neo-Hookean and Mooney–Rivlin models did not match any of the curvature of the reduced moduli. Similar trends to those described for PEGDA 2000 were observed for PEGDA with molecular weights of 575 (shown in the Supplementary Materials), 700 (shown in Figure 3), and 4000 Da (shown in Figure 5). However, the PEGDA at 575 and 700 Da showed a noticeably worse performance compared to the Rubinstein–Panyukov and Localization models, which consistently underpredicted the reduced stress at all concentrations except those of 25 wt% or greater. This is expected, as PEGDA 575 and 700 Da have a c* of 20 wt% and 18 wt%, respectively, and thus must go to much higher concentrations than PEGDA 2000 before chain interactions become significant.

Figure 6 shows the trends of the moduli and model parameters obtained from the fits of the models to the Mooney plots as a function of the PEGDA concentration and molecular weight. Specifically, the figure includes the parameters derived for the hyperelastic neo-Hookean and Ogden models (Figure 4A), the Localization and Rubenstein–Panyukov models (Figure 6B,C) and the Mooney–Rivlin model (Figure 6D,E). The neo-Hookean model (Figure 6A) is usually fit over a very limited range, and, as such, when fitting the entire stress–strain curve, which it does poorly on the Mooney plot, it did not yield a meaningful trend. The Mooney–Rivlin (Figure 6D,E) fit the stress–strain data better than the neo-Hookean model but also demonstrated no clear trend in the fitted parameters. This model also did not fit the Mooney plot very well, and the manner of the poor fit changed with concentration, as shown in Figure 4. As the concentration at synthesis increased, there was a corresponding increase in G in the neo-Hookean fit and the G_c_ values from both the Localization (Figure 6B,C) and Rubinstein–Panyukov models. This is expected because, in all three cases, this value represents the covalent contribution to the shear modulus. In the case of the Localization and Rubinstein–Panyukov models, G_c_ is calculated from the shear modulus over the initial strain range of 0% to 10% and has the same values as reported in Figure 2, due to the complexity of calculating a theoretical shear modulus based on the structure of PEGDA [9]. The G_e_ values of the Rubinstein–Panyukov model and the Localization model are the dominant contributors to the shear modulus in the low-wt% gels. They increase with polymer wt%. It should be noted, however, that the corresponding increase in G_c_ is greater than the G_e_ increase.

### 2.3. Discussion

This work utilizes PEGDA as a model material to examine the potential of different hyperelastic models in fitting stress–strain data for hydrogels and in evaluating their capacity to provide insight into the nature of deviations from the widely applied neo-Hookean model. While it is typically assumed that hydrogels behave as neo-Hookean solids due to the dilution of chains by swelling, which limits chain interactions that may cause strain-dependent effects, the results presented here indicate that two-parameter models better capture the full range of deformation behavior under compression. Additionally, the polymer mechanistic models indicate a greater role of entanglements than would be otherwise anticipated.

While no model would be expected to fit all data sets exactly, the models examined herein provide better fits than the standard neo-Hookean model by adding only a single additional parameter. Additionally, all models can be reduced to the neo-Hookean equation. In the literature, the second parameter in the Mooney–Rivlin, Ogden, and other models, such as Yeoh, is empirical; however, other models are derived using hypotheses about network interactions, including the Rubinstein–Panyukov and Localization models, among others. In this work, the Ogden model, which shares a functional form similar to the neo-Hookean model, provides a more accurate fit for higher strains across the broadest range of PEGDA formulations. However, many hydrogel parameters derived from the shear modulus are calculated under the assumption that hydrogels are neo-Hookean materials and the model is applied to limited strain range, as has been done extensively in the literature. The deviations from this standard model should be recognized when interpreting parameters such as the crosslink density or mesh size derived from the neo-Hookean model, or when correlating structure–property hypotheses with synthesis methodologies. This work has demonstrated that theory-derived models can be valuable tools for gaining a deeper understanding of the behavior of PEGDA or other hydrogels. However, the models do not capture the full range of behaviors that PEGDA may exhibit at different time and length scales. Poroelastic experiments using micro- and nanoindentation methods have been shown to be able to separate viscoelastic and poroelastic behavior, while the mechanistic models consider only elastic behavior [13,14,17,19,57].

The evaluation of hydrogel deformation only under uniaxial compression does not provide a comprehensive evaluation of the models or validate the generality of the results for other deformation modes [58,59]. However, focusing on the compression mode is significant in and of itself as it is the most challenging test for the models due to the need for compression data to capture a maxima when plotted on a “Mooney plot”, as in Figure 3, while the tensile mode is monotonic on such a plot [22]. Additionally, a focus on compression testing is important because this is the deformation mode of most importance in hydrogels in characterization and in applications [54,60].

#### 2.3.1. Two-Parameter Model Performance

As examined in the Section 2, each of the two-parameter models examined can recreate stress–strain data well over a broader range of strain than the neo-Hookean model, as shown in Figure 3. Deviations only appear at the upper end of the strain range. However, significant model differences are observed when the data are plotted in terms of reduced stress versus the inverse deformation ratio (a “Mooney plot”), which is better able to amplify the differences between the data and an ideal neo-Hookean solid, allowing for a more accurate evaluation of the models. The results obtained with two-parameter models are shown to correlate better with behavior than those obtained with the standard neo-Hookean model. The Mooney–Rivlin model accurately replicated the stress–strain behavior of the range of PEGDA formulations tested, especially at low concentrations of PEGDA at synthesis, as seen in Figure 4A–C. However, as the polymer concentration increased (Figure 4D,E), the shape of the reduced stress became more curved, which the Mooney–Rivlin model failed to capture. The limitation of the Mooney–Rivlin model is that a linear fit on the reduced stress plots cannot match non-linear deviations, at least for the two-term version of the Mooney–Rivlin model. While higher-order versions of the Mooney–Rivlin model do exist, they are usually restricted to a two-term model, and the parameters are further restricted to non-negative values. Furthermore, given their empirical nature, it is generally recognized that the values of the C_1_ and C_2_ parameters have a limited ability to explain the nature of the deviations [29].

In terms of accurately recreating the stress–strain curve and the reduced stress curve, the Ogden model yielded the best results. It outperformed any other tested model by accurately reproducing the behavior of the reduced stress across the entire strain range for the broadest range of formulations. This is due to the strain function of the model having an adjustable power dependence in α, which allows it to capture a wider range of curvatures than the other models. However, although it fits well and the two parameters from the model can be reduced to a shear modulus, it has been noted that α is experiment-dependent and not a measure of an intrinsic property of the material [61]. In the Ogden model, α is a measure of the non-linearity of the material, with values of α < 2 describing materials with a modulus that decreases as strain increases; α = 2 is of course the special case where the material is purely neo-Hookean, and α > 2 is when the modulus increases with increasing strain [62]. Furthermore, much like the Mooney–Rivlin model, the Ogden model is a power series that can be expanded to an arbitrary number of terms in order to increase the degree of accuracy of this model, although in this work we have limited it to only a single term and thus two parameters [63]. The biggest drawback to this model is that, much like the Mooney–Rivlin model, and in fact they share much of the same derivations, while it can be very accurate in reproducing data, it does not give further insight into the material network structure, nor is it noted to be very good at predicting stress–strain behavior outside the fitted data range [61]. In this case, while the Ogden model predicted a G generally matching the same order of magnitudes as the other models’ predictions for G, as seen in Figure 4, the parameters α and µ did not show any clear trend with increasing polymer concentration. This model falls short of the mechanistic models’ ability to provide additional insight into the hydrogel network structure; however, if the goal is to recreate the stress–strain behavior of a hydrogel accurately, the Ogden model is very useful.

The two-parameter mechanistic Rubinstein–Panyukov and Localization models have produced interesting results. The Rubinstein–Panyukov and Localization models yield similar results, as shown in Figure 3, Figure 4 and Figure 5. The trends in their derived parameters with formulation are the same, although the derived parameters differ in magnitude. This is due both to the models’ foundation in the Edwards tube model and their similar mathematical functions [64]. Both models generally fit the stress–strain curves over the entire tested range and capture the low-strain behavior in the reduced stress plots. However, as seen in Figure 4A,B, these models do not perform well in recreating the reduced stress behavior at mid-to-high strains when the polymer concentration is dilute, below the overlap concentration c*. This is likely due to the limited chain interactions in dilute solutions. At higher concentrations above c* (Figure 4C–E), these models performed much better, accurately capturing the features of the reduced stress plot. Changes in the properties of PEGDA relative to c* were also observed in a similar work by Tang et al. [9]. Another important limitation of these models is that both the Localization and the Rubenstein–Panyukov models predict an invariant maximum, occurring at λ ≈ 0.63 for the Localization model and λ ≈ 0.55 for the Rubenstein–Panyukov model. This maximum was an important feature of both models for capturing compressive behavior. While it has been shown to agree well with natural rubber, PDMS, and other elastomers, it does not always match the maxima seen in hydrogels. While the fact that they do have a similar curvature to the experimental data is promising, they do not decline as rapidly with inverse deformation as the data do.

#### 2.3.2. Physical Interpretation of Mechanistic Model Parameters

The mechanistic models differ significantly from the phenomenological models in that the derived parameters have a physical significance within the assumptions of the models from which they were derived. In Figure 6, both G_c_ and G_e_ increase with polymer concentration following consistent trends, unlike the phenomenological parameters. The parameter trends of both mechanistic models, Rubinstein–Panyukov and Localization, were very similar, though the Localization model gave around twice the value of G_e_ as the Rubinstein–Panyukov model. For both cases, while G_c_ increased by over 170 times as the polymer concentration went from 5% to 30%, G_e_ only increased around 20 times for PEGDA 2000 Da. Similar results and trends were seen with the other molecular weights examined As the polymer concentration increases, G_c_ is expected to increase more than G_e_, as it is known that polymers crosslinked below c* are more heterogeneous than those crosslinked above [9,47]. This heterogeneity is expected to lead to more deviations from pure neo-Hookean behavior, which G_e_ describes. As the concentration of the polymer approaches c*, the network is expected to become more homogeneous, and thus G_e_ relative to G_c_ should decrease. Additionally, as the polymer concentration increased above c*, when the uncrosslinked polymer chains began to overlap and entangle more (which, in turn, should increase G_e_), these models fit the data much more accurately than at lower concentrations. The improved goodness-of-fit aligns with the theory of chain interactions at this point, a key component of these mechanistic models. While these results hold with the theory, evaluating a broader range of polymer concentrations and other types of gels would be needed to confirm this interpretation of the deviations from neo-Hookean behavior.

Both of these models suggest that entanglements play a significant role in PEGDA gels, even at low strains. This allows the models to correlate the small-strain deformation trends that are often neglected when applying the neo-Hookean model, as seen in Figure 3. However, it is worth considering why PEGDA hydrogels may show a greater degree of entanglement contributions to the deformation response than would be anticipated by applying the neo-Hookean model to the same data set. It has been established that PEGDA hydrogels exhibit a significant degree of heterogeneity, with dense acrylate regions connected by sparse PEG chains. These dense regions around the acrylate backbones can lead to trapped and entangled chains, which can be a source of entanglements [65]. This trend agrees with the results of previously discussed experiments, and was also observed in the work by the Schultz group, which used Multiple Particle Tracking (MPT) to measure the stress relaxation behavior of the PEGDA hydrogel. MPT utilizes small probe molecules to measure the rheological properties of hydrogels on a microscopic scale [66]. The study found a significant difference in the polymer above and below the c* concentration. The PEGDA hydrogels formed below c* had both dissipative and elastic forces. When formed above c*, the PEGDA hydrogels were much more tightly crosslinked, and primarily elastic forces were present [67]. These results contradict the theoretical prediction that unpolymerized PEGDA solutions should not exhibit entanglements below 20 kDa. Furthermore, evidence from Padmavathi et al. suggests that PEGDA hydrogels at molecular weights as low as 200 Da exhibit some degree of entanglement [68]. This work found that higher molecular weights and concentrations led to stronger entanglements. They attributed this to PEGDA’s unique structure and hypothesized that the entanglements were not of the PEG chains themselves, which are thought to be too short to entangle, but of the acrylate backbone.

Although the hyperelastic models do not identify the exact nature of these entanglements, the results obtained from their use contribute to the idea that not only are entanglements present in PEGDA hydrogels, as has been hypothesized previously using different models, but they are also a significant contributor to their mechanical properties. While there have been multiple studies that corroborate the existence of entanglements in PEGDA hydrogels, further work is needed to consider the relationship between G_c_ and G_e_ in these models, which relates to other properties like the elastic storage modulus G’ and viscous loss modulus G” that could be found using dynamic mechanical analysis. The core difficulty in correlating the values of G_e_ with other related measurements of non-covalent chain interactions is that, while the concept of entanglements in networks is widely accepted as arising from topological constraints, their precise nature is interpreted differently according to different models, even though most are based upon the Edwards tube model (Rubenstein and Colby (p. 265), Erman and Mark [22,24]). Thus, correlating different measures of topological constraints is difficult [69]. Most techniques to quantify entanglements rely on a combination of indirect measurements and mathematical modeling, such as the work in this paper [70].

#### 2.3.3. Summary and Limitations

These results show that the two-parameter models identify deviations from neo-Hookean behavior in PEGDA hydrogels. Although the Mooney–Rivlin model did not provide additional insight or trends in the fitting of C_2_, the Localization model and Rubinstein–Panyukov model revealed a distinct trend with G_e_. Specifically, the resultant G_e_ value increases as the prepolymer wt% increases. While both models have different values for G_e_, they follow the same linear trend. This magnitude of entanglement contribution may be surprising. Still, prior studies have suggested the possibility of such contributions in other networks to which these models have been applied [38,44] and in PEGDA specifically [9]. Thus, the use of the Localized and Rubinstein–Panyukov models adds to the evidence that entanglements in PEGDA hydrogels are key contributors to their mechanical properties, as hypothesized in other studies.

It is worth noting that nearly all hyperelastic models for polymers were initially developed for solid elastomers rather than highly swollen hydrogels. Therefore, a simple swelling correction in line with other commonly reported swelling correction factors was employed when fitting the data in this work [38]. While many models have subsequently incorporated a swelling correction factor, it is generally expected that, as gels become highly swollen, they will exhibit a more neo-Hookean behavior [43]. A shortcoming of these swelling correction factors is that they only consider the degree of swelling of the hydrogel at testing conditions, and not the polymer volume fraction at the formation conditions [41]. This is an important consideration because the formation state has been shown to impact the resultant mechanical properties. The most widely used correction for the difference between the network at synthesis (relaxed state) and at swelling equilibrium was developed in 1976 by Peppas and Merrill, and was recently modified by Richbourg and Peppas; this scales the swollen state relative to the relaxed state in each elastic term [6,71], as it scales each of the models in the same way, and thus would affect the magnitudes of the terms but not the trends. Additionally, for PEGDA, the gels swell very little from the formation state, so that a molecular weight effect would not be expected either. Thus, we do not expect that it would alter the trends, fittings, or conclusions of this work [6]. However, accounting for the volume fraction at formation could be accounted in extensions of this work [43].

Another limitation of the mechanistic models used in this work is that, while they fit the data and are based on the generally accepted idea of entanglements as topological constraints on chain deformation, directly correlating their values with independent measurements would not be straightforward, unlike accounting for covalent crosslinking by different methods [70].

While the models presented in this paper demonstrated the ability to fit PEGDA data, they were originally developed for solid elastomers, and they are scaled according to a chain dilution factor that has long been used. A key difference between solid elastomers and hydrogels is the presence of a solvent, in this case water, in the network. Based on the prior literature and our own measurements and observations, there is a negligible migration of the solvent during the time scale of such experiments, and thus the poroelastic effects have not been considered [8,9,16]. However, the role of poroelastic effects and solvent migration might help explain the discrepancies between our data and the models presented and might be considered for incorporation into those models.

## 3. Conclusions

This study found that two-parameter hyperelastic models describe the deformation behavior of PEGDA, a widely studied hydrogel, under uniaxial compression much better than the commonly used neo-Hookean model. The models examined were loosely classified into two categories: phenomenological models, which add a second empirical parameter to capture strain dependence, and mechanistic models, which add a second parameter derived from theoretical models of how network chains interact under stress. The models were compared in terms of their ability to replicate the stress–strain curves directly and by the more revealing plots of reduced stress against the inverse deformation ratio (“Mooney plots”). The uniaxial compression of PEGDA gels of several different molecular weights of PEGDA precursors over a wide synthesis concentration range of formulations was examined. When the full strain range up to fracture was examined, all formulations displayed a significant deviation from neo-Hookean behavior. All of the two-parameter models were able fit the stress–strain data. However, the Mooney plots showed that the nature of the deviation changes significantly with the composition but not with the molecular weight.

In terms of fitting the stress–strain curves to higher strains on the Mooney plots, the phenomenological Ogden model provides the best fit for the range of PEGDA formulations tested out of all the phenomenological and mechanistic models. The Mooney–Rivlin model, the most widely known two-parameter model, was able to fit the stress–strain curves, but could not capture the observed features of the Mooney plots, most notably the compression maxima.

The mechanistic Localization and Rubinstein–Panyukov models separate the resistance to deformation into modulus contributions from crosslinked chains, G_c_, and entanglements, G_e_. Both fit the Mooney plots for gels synthesized at concentrations above the overlap concentration c*, but not below it, which is consistent with the concepts upon which the models were built. Above c*, however, these models accurately capture the small-strain behavior and the curvature and maxima in these plots at higher strains. Thus, these mechanistic models performed better at the higher molecular weights for the same concentrations of PEGDA since c* is a function of molecular weight, with higher molecular weights leading to a lower c*. The entanglement parameter G_e_ was a significant contributor to deformation response for all of the gels tested, and this led to the ability of the model to capture small-strain behavior that the neo-Hookean model did not for these gels. The trends in G_e_ and G_c_ with synthesis concentration indicated that, as the polymer solution became more concentrated, the shear modulus contribution from the network crosslinks became much more significant, becoming equal to or greater than the contribution from entanglements. The existence of entanglements in PEGDA gels had been suggested in prior PEGDA studies due to the complex structure of the polyacrylate kinetic changes and the PEG linkers. The magnitude of entanglement contribution suggested by these models is notable and requires further study to validate the assumptions upon which it is built. However, the fact that these models are built on a theory of entanglement mechanics and can accurately fit the mechanical data under specific circumstances implies that the underlying assumptions and theories are likely valid. Further study is underway to confirm the validity of this interpretation, both for PEGDA of a broader range of formulations and also different classes of gels as biopolymers crosslinked by different methods, where the entanglement contribution in a solution is more directly linked to the entanglements found in the crosslinked gels.

Modeling the stress–strain behavior of elastomers with various hyperelastic models has been extensively studied for decades. However, for hydrogels—water-swollen elastomers—it has been widely assumed that chain dilution upon swelling would lead to neo-Hookean behavior, and this has been generally accepted as valid among hydrogel researchers, especially those working with gels for biomedical applications, although the validity of the assumption is infrequently tested. The neo-Hookean model has been used almost exclusively to obtain the shear moduli of hydrogels from deformation data, typically in uniaxial compression, to calculate network parameters such as crosslink density, molecular weight between crosslinks and network mesh size. Thus, we believe that this study shows the value of using two-parameter models for capturing deviations from neo-Hookean behavior. Also, we believe that these models can help in identifying the origin of the deviations from the neo-Hookean model and can provide greater insight into the nature of the chain interactions that determine the deformation response of the gel.

The neo-Hookean model is expected to be useful for many hydrogels, particularly those that are more highly swollen than PEGDA. Although this work examined only PEGDA, it demonstrates the ability of two-parameter models to replicate the stress–strain curve of hydrogels over broader strain ranges than the neo-Hookean model. Additionally, the polymer mechanistic models demonstrate their potential to explain the origin of deviations in terms of network structure and interactions.

## 4. Materials and Methods

### 4.1. Sources of Materials and Software

Poly(ethylene glycol) diacrylate (PEGDA; Mn = 575 (10 repeat PEG units); M_n_ = 700; CAS = 26570-48-9 (13 repeat PEG units); M_n_ = 2000 Da; CAS = 26570-48-9 (39 repeat PEG units); and M_n_ = 4000 Da; CAS = 26570-48-9 (85 repeat PEG units)) were Sigma Aldrich brand. Lithium phenyl-2,4,6-trimethylbenzoylphosphinate (“LAP” >95% purity; CAS = 85073-19-4) was purchased from MilliporeSigma, TCI Chemicals brand. Deionized ultra-filtered water (“DIUF”, CAS = 7732-18-5) was ThermoFisher Scientific (Waltham, MA, USA) brand. All materials and reagents were used as received.

The Ogden model analysis was performed with the assistance of a publicly available custom MATLAB^®^ (Version R2025b) script developed by Emi Kiyotake [3]. All other modeling was done utilizing Microsoft Excel (Office 16).

### 4.2. Methodology

The key steps in the methodology are the synthesis of the hydrogels, determination of the swelling degree, the uniaxial compression measurements and the modeling. These are each described in the following subsections.

#### 4.2.1. Synthesis of PEGDA Hydrogels

PEGDA hydrogels were prepared as aqueous solutions with varying initial PEGDA weight percentages (wt%) ranging from 5 to 30 wt% and containing 0.005 g/g LAP photoinitiator, as it has been shown to be an effective photoinitiator and more soluble than other commonly used commercial options, such as the commonly used irgacure 2959 [72]. Solutions were briefly vortexed to ensure complete dissolution and subsequently bubbled with nitrogen for 5 min to saturate the solution with nitrogen, thereby displacing oxygen and preventing oxygen inhibition of the polymerization reaction. Next, the PEGDA was pipetted into a mold made with two glass slides separated by a 2 mm rubber gasket. The hydrogel was formed through photopolymerization in a Spectrolinker UV crosslinker (Spectro-UV, Farmingdale, New York, USA) at 312 nm, with an irradiation intensity of 3.15 mW/cm^2^ for 500 s on each side of the mold, as this wavelength has been shown to have a strong molar extinction coefficient for LAP [72]. After gelation, the hydrogel strip was removed from the molds, and PEGDA disks were made using a 5 mm biopsy punch. These samples were placed in DIUF water, and the water was changed every 12 h for a minimum of 72 h total to leach out any unreacted components and for the gels to reach swelling equilibrium.

#### 4.2.2. Determination of Swelling Degree

The hydrogel disks swollen to equilibrium were gently blotted with Kimwipes^®^ (Kimberly-Clark Professional, Irving, TX, USA) to remove surface water, and the swollen mass was determined using an analytical balance. Subsequently, the disks were placed inside a desiccation chamber over CaSO_4_ desiccant at ambient temperature until a constant dry mass was reached. The hydrogels’ mass swelling degree (Q) was defined as the ratio of the swollen mass to the dry mass. Densities were then used to calculate a volumetric swelling degree through conventional methods.

#### 4.2.3. Uniaxial Compression Measurements

The mechanical analysis procedure followed our previously published work [33,54]. The unconfined, uniaxial compression of PEGDA disks was performed using an RSA III dynamic mechanical analyzer (TA Instruments, New Castle, DE, USA) at a range of strain rates from 0.1 mm/s to 0.0025 mm/s. From this, it was determined that 0.005 mm/s is sufficiently slow to minimize strain rate-dependent effects. The compression plates were lubricated with mineral oil, and no barreling was observed during the compression process. The sample diameter was measured using a micrometer (Mitutoyo America Corporation, City of Industry, CA, USA) viewed under a stereomicroscope (~10× magnification, Olympus, Center Valley, PA, USA).

#### 4.2.4. Modeling

Here, the methods used to fit the hyperelastic models to the data to obtain the parameters reported in the Section 2 are presented. The hyperelastic models were originally developed for solvent-free elastomers, but each has been modified to account for the dilution of the chains by swelling, thereby converting the modulus to an equivalent dry basis. The exception is the Rubinstein–Panyukov model, so it was modified using the method published for the similar Localization model. Presented in Equations (7)–(11) are the modified equations of the reduced stress (Equation (7)) [73], the Mooney–Rivlin (Equation (8)) [30], the Rubinstein–Panyukov (Equation (9)), and the Localization models (Equation (10)) [38] used to fit the stress–strain data for the hydrogels. Equation (11) represents the neo-Hookean model in terms of the reduced stress.

(7) [f*]=σλ−λ−2φ13

(8) [f*]=2(C1+φ13C2λ)

(9) [f*]=Gc+φ13Ge1−λ(−32)(λ−λ−2)

(10) [f*]=Gc+φ13Ge0.74λ+0.61λ−0.5−0.35

(11) $$[f^{*}]=G$$

Note that the value of G from the neo-Hookean model reported in most published studies is not corrected for swelling as it has been in this work. Rather, it is found using the reduced stress, as given in Equation (2), rather than Equation (7), as in this work. The neo-Hookean G has been corrected for swelling in this work to compare all models on the same basis.

Thus, the parameters in the different models were obtained by fitting the model to the data on a plot of reduced stress [f*] against 1/λ (a “Mooney plot”), as this has been demonstrated to be effective in identifying the magnitude of a material’s deviations from neo-Hookean behavior and thus the ability of the models to accurately capture that deviation [30,38,41]. A benefit of the Mooney plot is that it is more sensitive to deviations from neo-Hookean behavior than a stress–strain plot [30]; thus, its form tends to be better for fitting models. This enhanced sensitivity is partly due to keeping deviations at high strains from overwhelming deviations at small strains. Another advantage of a Mooney plot is that the large deformation in the geometry of the sample itself causes some of the deviations from the neo-Hookean model. Normalizing the stress by (λ − 1/λ^2^) allows for this geometry distortion to be partially corrected [30].

Therefore, the parameters for the neo-Hookean, Mooney–Rivlin, Localization, and Rubinstein–Panyukov models were found by fitting them to Mooney plots. For the neo-Hookean and Mooney–Rivlin models, the adjustable parameters were selected to maximize the R^2^ value of the predicted model value compared to the experimental data over the entire stress–strain range. It should be noted that, in the hydrogel literature, the neo-Hookean model is usually fitted over the strain range where a plot of stress vs. (λ − 1/λ^2^) is adequately linear in the authors’ judgment. This is done in recognition that the assumptions in the neo-Hookean model break down at higher strains, such as the assumption of a Gaussian distribution of chain lengths at all extensions. Here, the neo-Hookean model was fitted to the entire strain range in this work to compare its performance at higher strains with that of the two-parameter models. The Localization model and Rubinstein–Panyukov model were fitted in a similar manner. However, the G_c_ for these models is ideally calculated from a polymer’s theoretical crosslink density based on stoichiometry. However, PEGDA’s complex network structure, comprising diacrylate kinetic chains and PEG linkers, makes this approach difficult to apply. Therefore, we elected to assume that the value of G_c_ was equivalent to the shear modulus from the initial portion of the stress–strain curve, consistent with model assumptions. Once G_c_ was obtained, G_e_ was calculated by fitting the Localization and the Rubinstein–Panyukov models to the Mooney plot. A G_e_ that maximized the R^2^ value between the model’s predicted value and the experimental data was obtained.

For the Ogden model, the published program was used to fit the model to the stress–strain curve and obtain values of the material constant, µ, and the non-affine parameter, α. These values were then employed to determine a shear modulus based on the relationship G = µα/2 as derived in Ogden’s original work [25]. To compare the model’s trends to the others on the reduced stress, the stress–strain predicted by the µ and α was generated by the model and replotted as [f*] vs. 1/λ.

## Data Availability

The raw data supporting the conclusions of this article will be made available by the authors on request.

## Supplementary Materials

The following supporting information can be downloaded at: https://www.mdpi.com/article/10.3390/gels12020171/s1, Figure S1: Stress-strain graphs for PEGDA 575 Da; Figure S2: Reduced stress graphs for PEGDA 575 Da; Figure S3: Stress-strain graphs for PEGDA 700 Da; Figure S4: Reduced stress graphs for PEGDA 700 Da; Figure S5: Stress-strain graphs for PEGDA 2000 Da; Figure S6: Reduced stress graphs for PEGDA 2000 Da; Figure S7: Stress-Strain graphs for PEGDA 4000 Da; Figure S8: Reduced stress graphs for PEGDA 4000 Da; Figure S9: Flory-Huggins solubility parameter for PEGDA; Figure S10: Crosslink density of various molecular weights of PEGDA calculated using the small strain neo-Hookean model; Table S1: PEGDA 575 Da Modeling parameters; Table S2: PEGDA 700 Da modeling parameters; Table S3: PEGDA 2000 Da modeling parameters; Table S4: PEGDA 4000 Da modeling parameters; Table S5: Overlap concentration of PEGDA.

## Abbreviations

The following abbreviations are used in this manuscript:

PEGDA	Poly(ethylene glycol) diacrylate
PDMS	Polydimethylsiloxane
LAP	Lithium phenyl -2,4,6-trimethylbenzoylphosphinate
PHA	Pentenoate-modified hyaluronic acid
SANS	Small-angle neutron scattering
TD-NMR	Time-domain nuclear magnetic resonance
SAXS	Small-angle X-ray scattering
