# Supplementary material for: Characterization of Hydrogel Deformation Using Two-Parameter Hyperelastic Models
Source: Gels. 2026 Feb 14;12(2):171. doi: 10.3390/gels12020171 (PMC12940749; doi:10.3390/gels12020171)
Supplement: Supplementary file 1 [file gels-12-00171-s001.zip › gels-4108417-supplementary.pdf]

Use of Two-Parameter Hyperelastic Models for Mechanical Characterization of Hydrogels

Joseph M. Scalet (jmscalet@ku.edu), Faiz Mandani, Stevin H. Gehrke (shgehrke@ku.edu)

The stress-strain (Figure S.1) and reduced stress (figure S.2) graphs of Poly(ethylene Glycol Diacrylate) (PEGDA) 575 Da along with the other molecular weights of PEGDA, 700 Da (figures S.3 and S.4), recreations of the PEGDA 2000 Da plots found in the main body of this manuscript (figures S.5 and S.6), and 4000 Da (figures S.7 and S.8) are provided as they were omitted from the main body due to size and space constraints.

Figure S.9 shows the Flory-Huggins solubility parameter calculated from Flory-Rehner theory, indicating a linear trend of increasing χ with increasing polymer weight percentage.

Figure S.10 shows the calculated crosslink density based on the small strain neo-Hookean model

The predicted moduli for each wt% of the discussed models for PEGDA 575 DA (Table S.1), 700 Da (Table S.2), 2000 Da (Table S.3), and 4000 Da (Table S.4) are presented.

Finally, Table S.5 lists the c*, overlap concentration, of PEGDA for the utilized molecular weights.

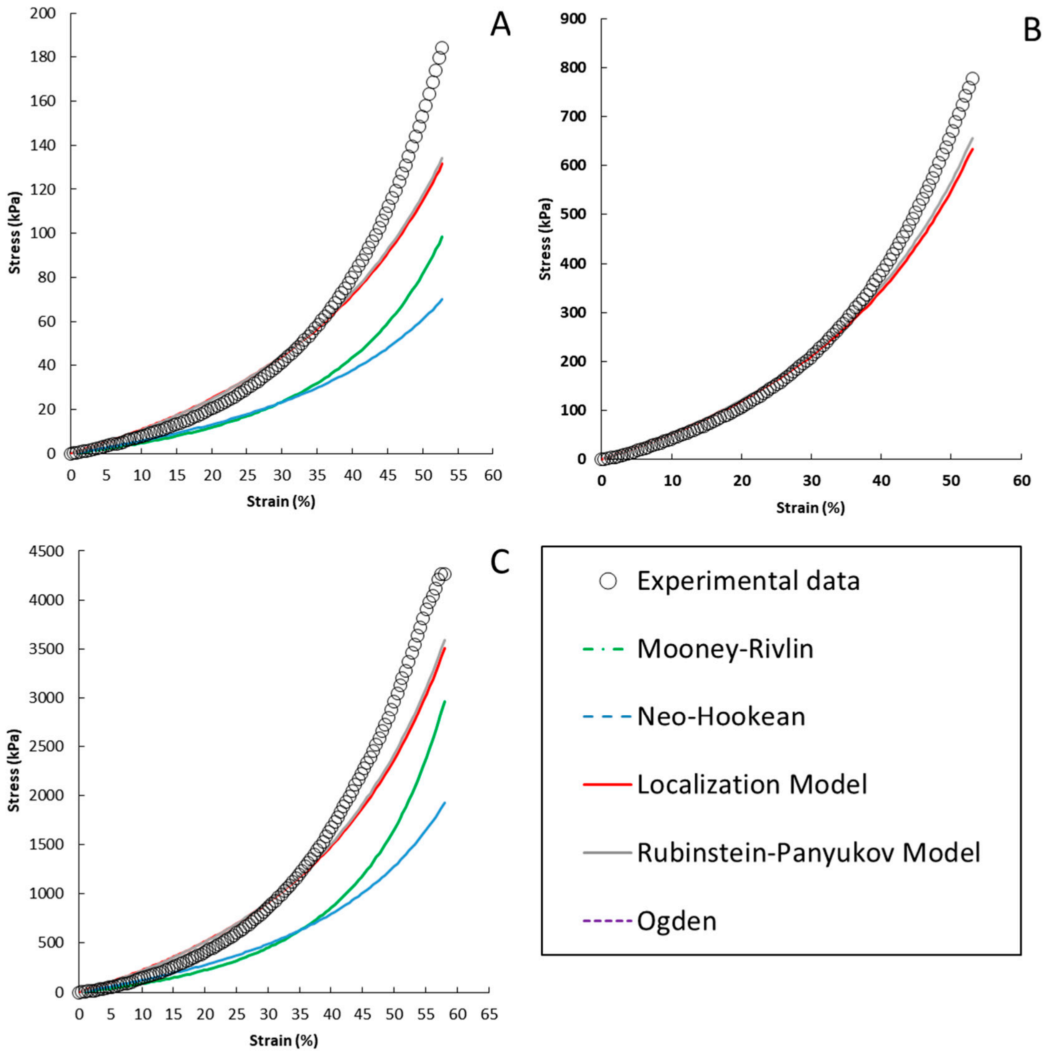

Figure S1. - Stress-strain graphs for PEGDA 575 DA at a polymer at A) 10 wt% B) 15 wt% C) 20 wt%

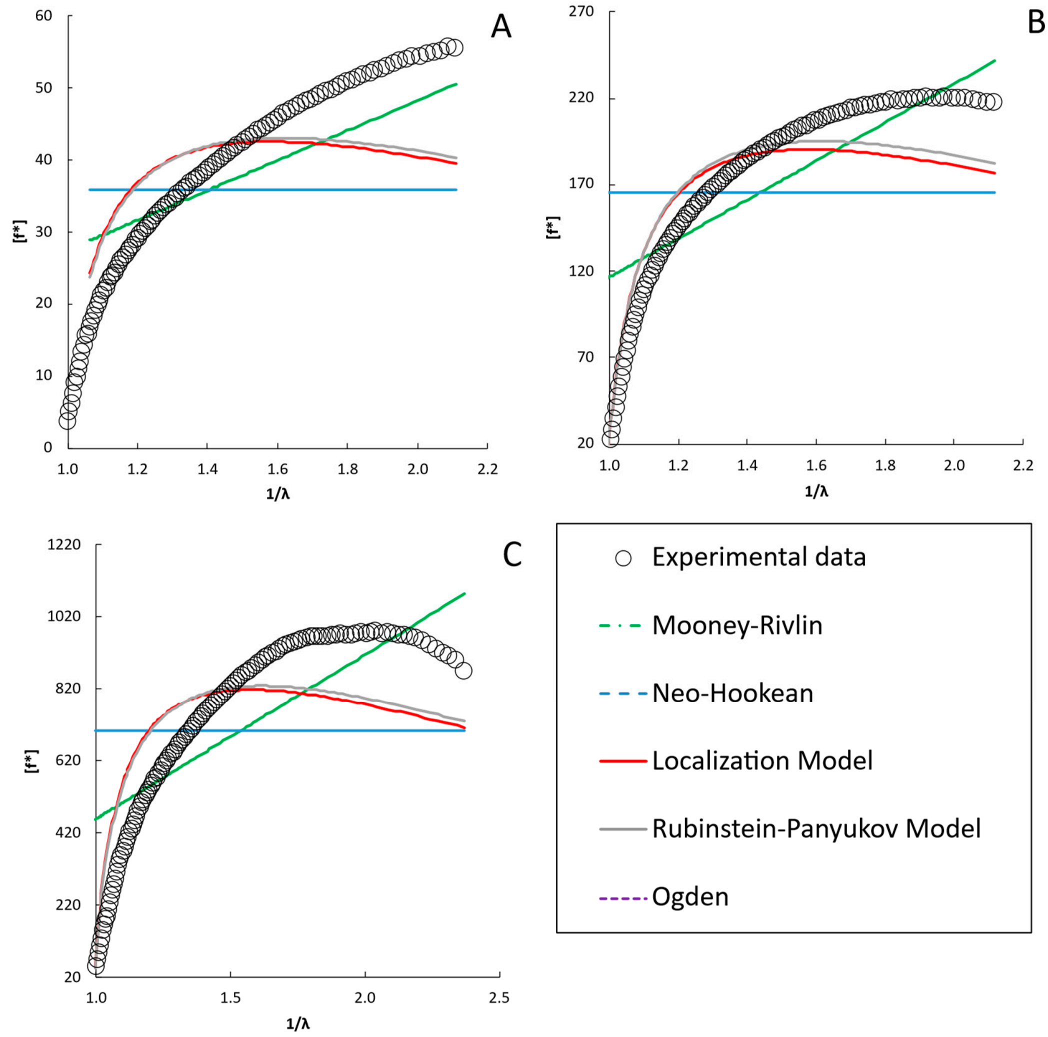

Figure S2. - Reduced stress graphs for PEGDA 575 DA at a polymer at A) 10 wt% B) 15 wt% C) 20 wt%

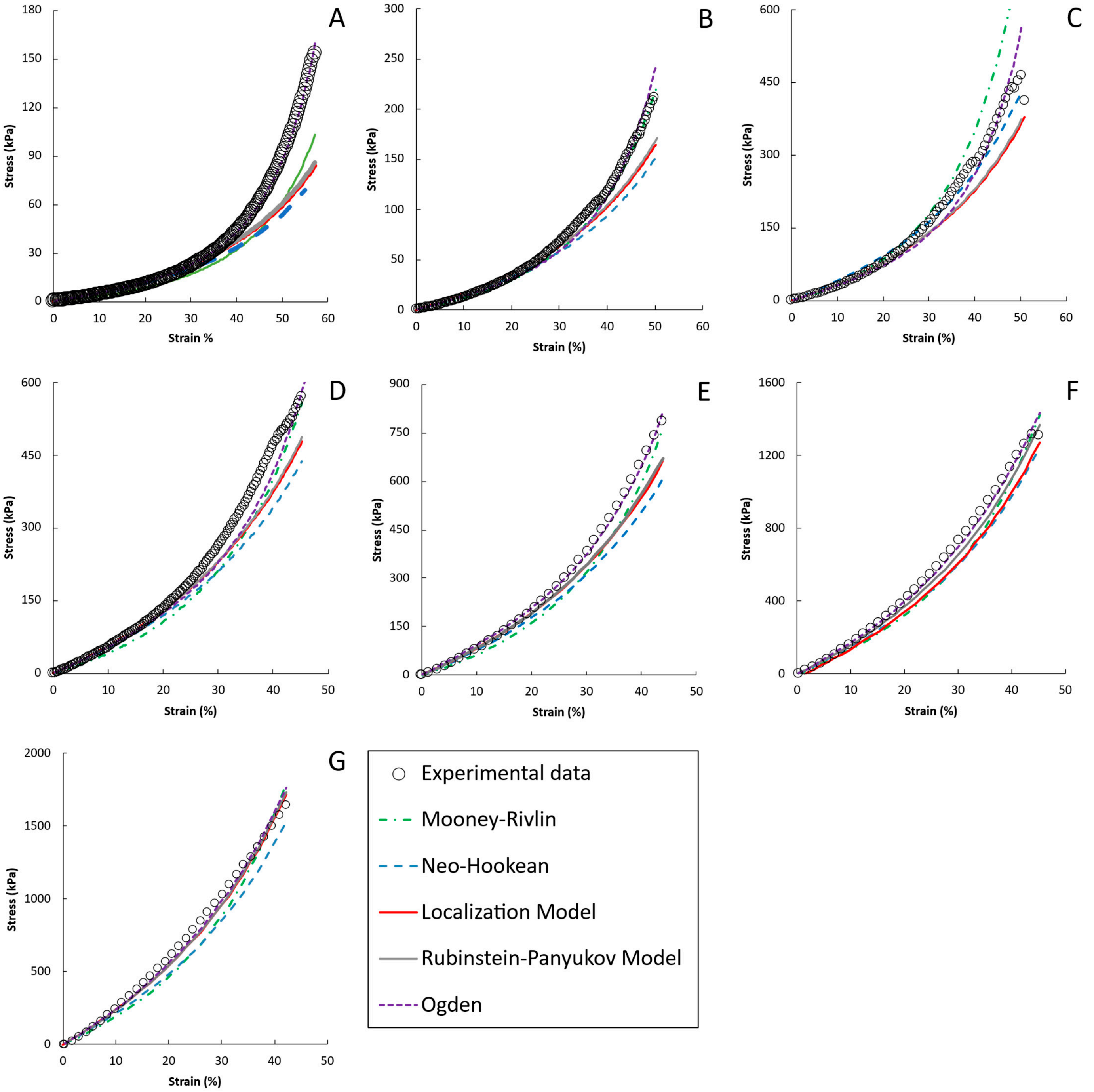

Figure S3. – Stress-strain graphs for PEGDA 700 DA at a polymer at: A) 10 wt%, B) 12 wt%, C) 15 wt%, D) 17 wt%, E) 20 wt%, F) 25 wt%, and G) 30

wt%

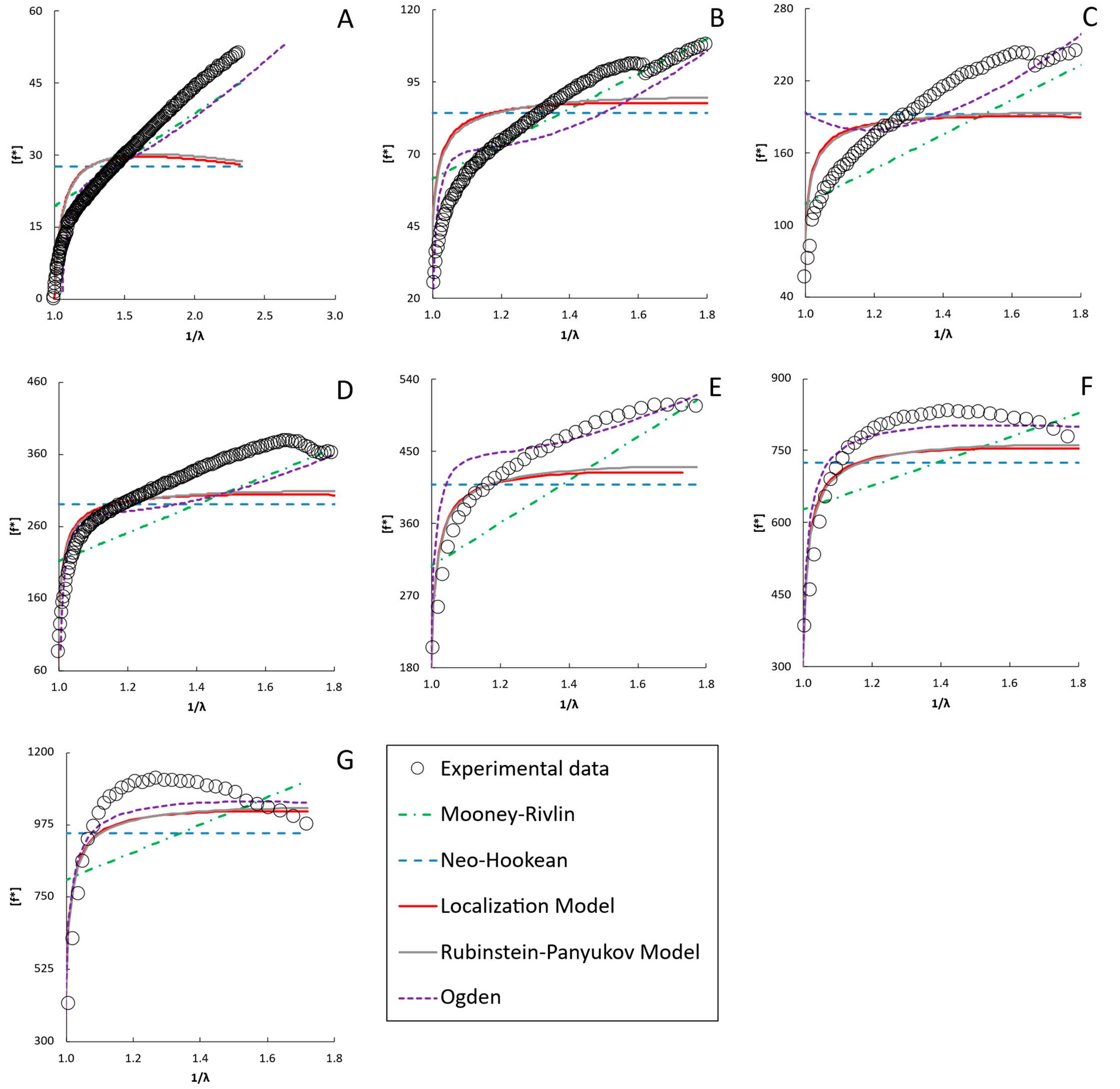

Figure S4.– Reduced stress graphs for PEGDA 700 DA at a polymer at: A) 10 wt%, B) 12 wt%, C) 15 wt%, D) 17 wt%, E) 20 wt%, F) 25 wt%, and G) 30 wt%

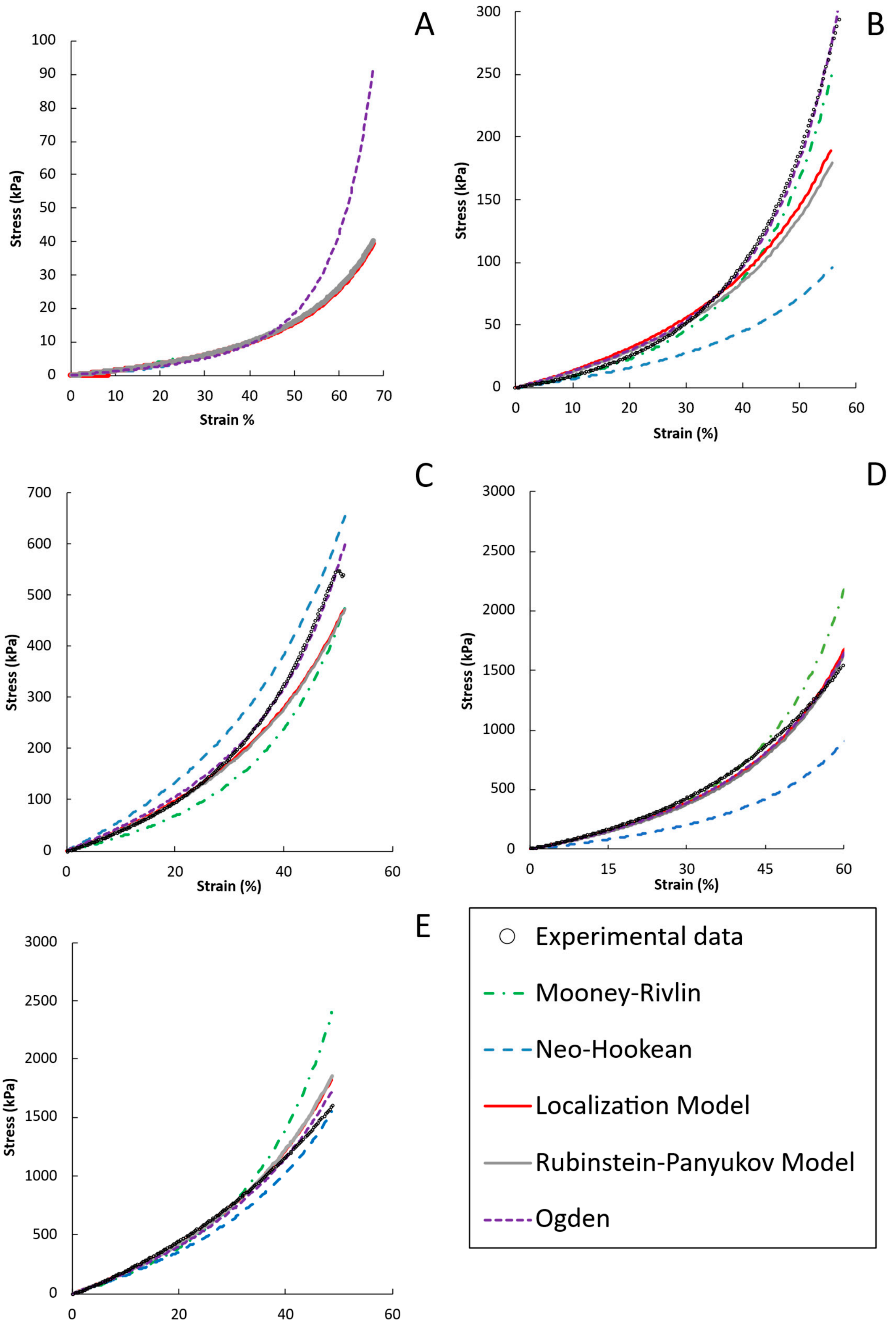

Figure S5. - Stress-strain graphs for PEGDA 2000 DA at a polymer at A) 5 wt% B) 10 wt% C) 15 wt% D) 20 wt% E) 30 wt%

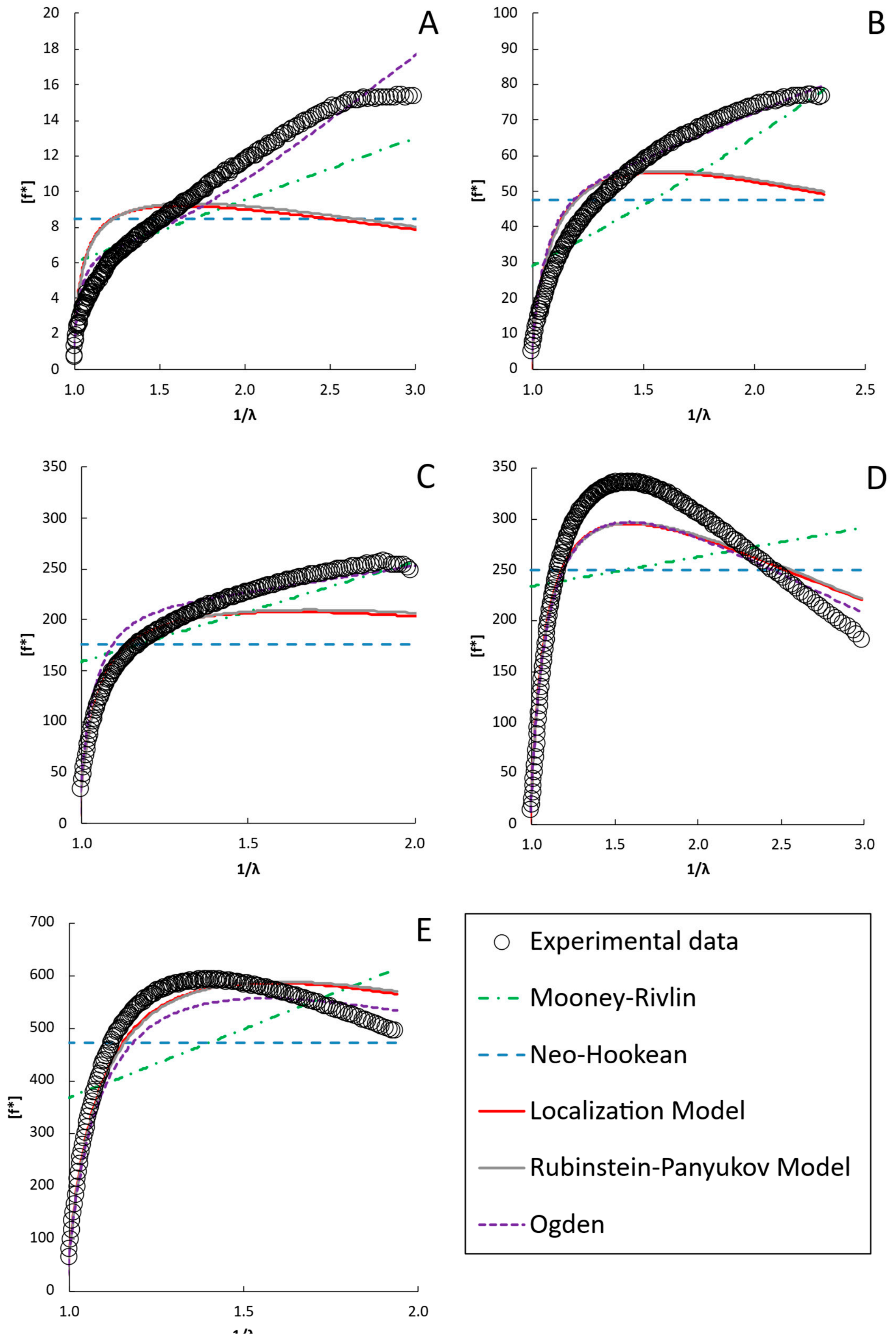

Figure S6.– Reduced stress graphs for PEGDA 2000 DA at a polymer at A) 5 wt% B) 10 wt% C) 15 wt% D) 20 wt% E) 30 wt%

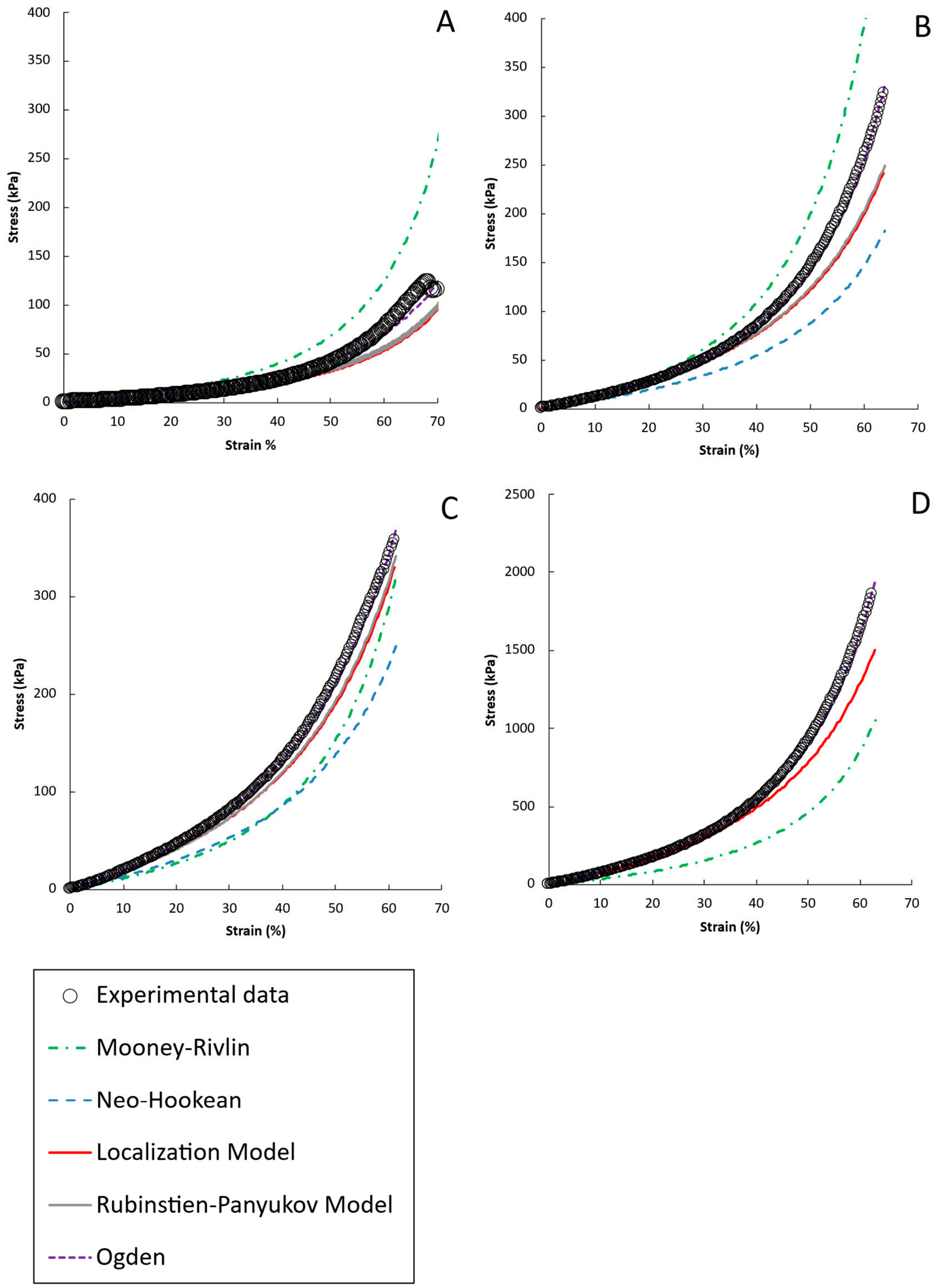

Figure S7. – Stress-Strain graphs for PEGDA 4000 DA at a polymer at: A) 7 wt%, B) 10 wt%, C) 12 wt%, and D) 30 wt%.

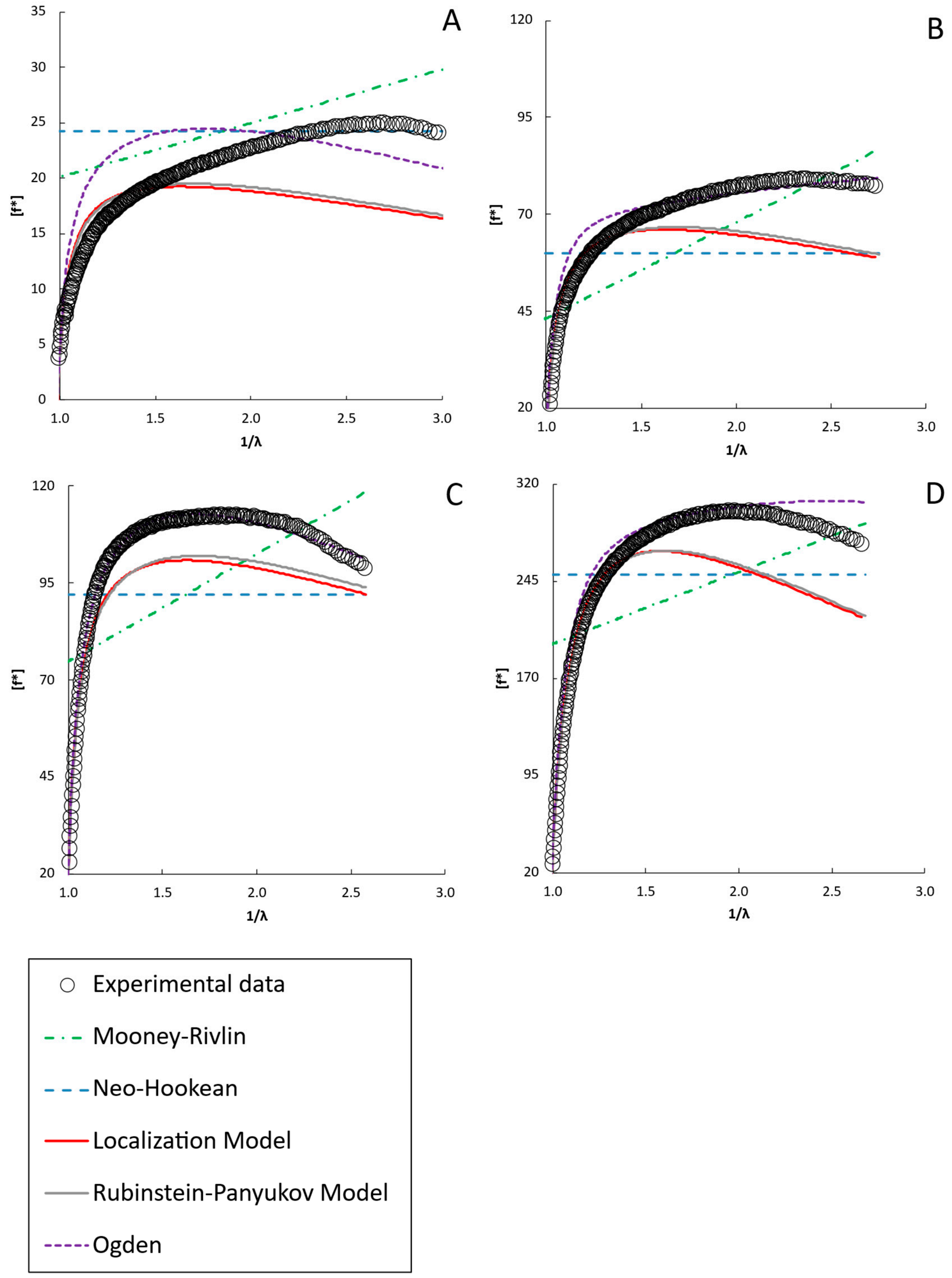

Figure S8. – Reduced stress graphs for PEGDA 4000 DA at a polymer at: A) 7 wt%, B) 10 wt%, C) 12 wt%, and D) 30 wt%.

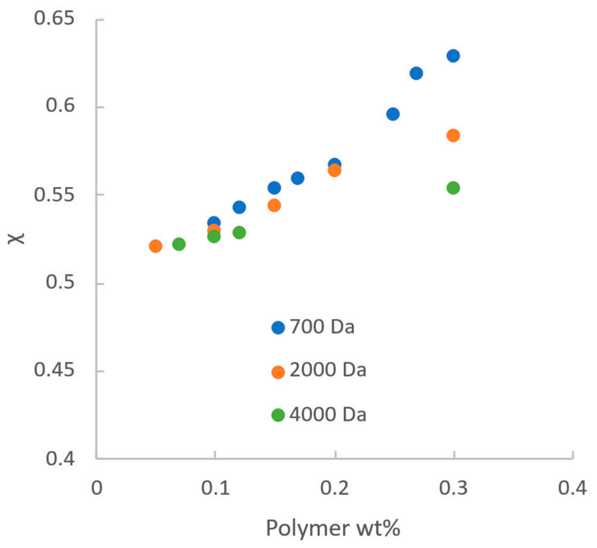

Figure S9. - Flory-Huggins solubility parameter for PEGDA. All molecular weights exhibited a linear trend with concentration aligning with previously reported literature.

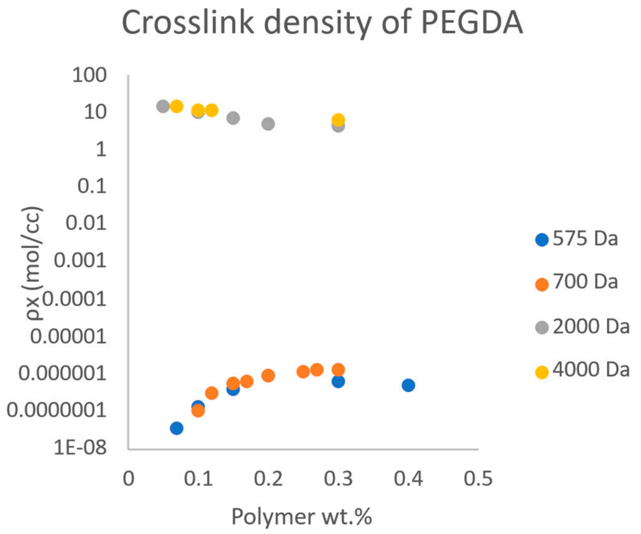

Figure S10. - Crosslink density of various molecular weights of PEGDA calculated using the small strain neo-Hookean model

Table S1.- PEGDA (575 Da) Modeling parameters

PEGDA (575 Da) modeling parameters

Wt%.	NeoHookean G Avg	± StDev	Mooney-Rivlin $2C_1$ Avg	± StDev	Mooney-Rivlin $2C_2$ Avg	± StDev	Rubinstein-Panyukov G_c Avg	± StDev	Rubinstein-Panyukov G_e Avg	± StDev	Localization Model G_c Avg	± StDev	Localization Model G_e Avg	± StDev	Ogden Model G Avg	± StDev	Ogden Model α Avg	± StDev
10%	34.78 ±	1.12	37.44 ±	89.29	77.82 ±	24.75	29.73 ±	1.03	63.14 ±	3.35	29.73 ±	1.03	135.01 ±	7.03	31.77 ±	0.79	3.80 ±	2.65
15%	152.8 ±	14.0	25.3 ±	40.8	169.1 ±	73.4	144.9 ±	9.7	255.8 ±	22.4	144.9 ±	9.7	536.5 ±	42.1	137.4 ±	13.2	0.26 ±	0.27
20%	617.7 ±	27.7	617.7 ±	27.8	0.0 ±	0.0	622.0 ±	184.1	1130.4 ±	27.7	622.0 ±	184.1	2402.7 ±	58.3	630.1 ±	99.0	0.69 ±	0.27

Wt%.	NeoHookean R^2 Avg	± StDev	Mooney-Rivlin R^2 Avg	± StDev	Rubinstein-Panyukov R^2 Avg	± StDev	LM R^2 Avg	± StDev	Ogden* R^2 Avg	± StDev	Swelling (v/v) Q Avg	± StDev
10%	0.04 ±	0.02	0.55 ±	0.00	0.63 ±	0.04	0.66 ±	0.02	0.81 ±	0.02	9.8 ±	0.0
15%	0.06 ±	0.04	0.54 ±	0.20	0.77 ±	0.09	0.74 ±	0.09	0.67 ±	0.37	8.8 ±	0.0
20%	0.37 ±	1.73	0.03 ±	0.05	0.50 ±	0.07	0.50 ±	0.06	0.74 ±	0.36	7.2 ±	0.2

Table S2. - PEGDA (700 Da) Modeling parameters

PEGDA (700 Da) modeling parameters

Wt%.	NeoHookean G Avg	± StDev	Mooney-Rivlin $2C_1$ Avg	± StDev	Mooney-Rivlin $2C_2$ Avg	± StDev	Rubenstien-Panyukov G_c Avg	± StDev	Rubenstien-Panyukov G_e Avg	± StDev	Localization Model G_c Avg	± StDev	Localization Model G_e Avg	± StDev	Ogden Model G Avg	± StDev	Ogden Model α Avg	± StDev
0.1	26.5 ±	1.1	0.0 ±	0.0	20.4 ±	0.8	14.7 ±	0.6	42.4 ±	1.8	14.7 ±	0.6	88.4 ±	3.8	17.6 ±	1.5	5.40 ±	0.49
0.12	90.9 ±	7.5	0.0 ±	0.0	64.5 ±	5.2	43.4 ±	2.6	97.9 ±	10.5	43.4 ±	2.6	203.6 ±	22.2	45.3 ±	2.9	6.69 ±	0.50
0.15	201.2 ±	12.2	-15.3 ±	4.9	149.8 ±	18.5	109.3 ±	13.2	173.2 ±	6.3	109.3 ±	13.2	385.7 ±	16.6	109.9 ±	8.0	6.30 ±	0.85
0.17	271.7 ±	17.1	-20.9 ±	27.5	210.4 ±	29.7	174.6 ±	6.8	208.6 ±	18.8	174.6 ±	6.8	463.8 ±	13.1	187.2 ±	12.7	4.76 ±	0.79
0.2	437.1 ±	27.1	8.8 ±	8.6	360.4 ±	109.0	287.5 ±	4.8	248.7 ±	17.1	287.5 ±	4.8	623.5 ±	80.1	296.3 ±	5.4	3.98 ±	0.29
0.25	704.8 ±	17.6	120.2 ±	58.7	280.1 ±	67.7	539.3 ±	17.4	351.5 ±	11.2	539.3 ±	17.4	771.1 ±	53.0	498.3 ±	8.7	1.38 ±	0.19
0.27	927.7 ±	22.9	395.5 ±	118.1	80.5 ±	131.5	659.5 ±	18.7	382.5 ±	23.3	659.5 ±	18.7	786.8 ±	56.8	632.4 ±	17.4	1.91 ±	0.14
0.3	955.6 ±	10.6	164.7 ±	155.7	366.5 ±	196.3	808.9 ±	29.2	367.9 ±	37.7	808.9 ±	29.2	788.2 ±	68.5	756.8 ±	54.1	2.26 ±	0.77

Wt%.	NeoHookean R^2 Avg	± StDev	Mooney-Rivlin R^2 Avg	± StDev	Rubenstien-Panyukov R^2 Avg	± StDev	LM R^2 Avg	± StDev	Ogden* R^2 Avg	± StDev	Swelling (v/v) Q Avg	± StDev
0.1	0.02 ±	0.01	0.22 ±	0.01	0.48 ±	0.01	0.46 ±	0.01	1.00 ±	0.00	10.6 ±	0.0
0.12	0.10 ±	0.05	0.85 ±	0.03	0.49 ±	0.04	0.49 ±	0.06	0.99 ±	0.00	8.6 ±	0.0
0.15	0.18 ±	0.01	0.62 ±	0.10	0.58 ±	0.02	0.61 ±	0.06	0.96 ±	0.02	7.0 ±	0.2
0.17	0.13 ±	0.02	0.65 ±	0.04	0.66 ±	0.03	0.68 ±	0.05	0.98 ±	0.01	6.4 ±	0.1
0.2	0.54 ±	0.04	0.58 ±	0.11	0.78 ±	0.03	0.85 ±	0.02	0.99 ±	0.01	5.7 ±	0.0
0.25	0.23 ±	0.01	0.46 ±	0.09	0.86 ±	0.01	0.89 ±	0.01	1.00 ±	0.00	4.3 ±	0.0
0.27	0.48 ±	0.15	0.32 ±	0.19	0.60 ±	0.02	0.59 ±	0.06	1.00 ±	0.00	3.6 ±	0.0
0.3	-0.05 ±	0.05	0.44 ±	0.03	0.86 ±	0.10	0.87 ±	0.07	1.00 ±	0.00	3.4 ±	0.0

Table S3. - PEGDA (2000 Da) Modeling parameters

PEGDA (2000 Da) modeling parameters

	**NeoHookean**		**Mooney-Rivlin**				**Rubenstien-Panyukov**				**Localization Model**				**Ogden***			
	G		**$2C_1$**		**$2C_2$**		G_c		G_e		G_c		G_e		G		α	
Wt%.	Avg	± StDev	Avg	± StDev	Avg	± StDev	Avg	± StDev	Avg	± StDev	Avg	± StDev	Avg	± StDev	Avg	± StDev	Avg	± StDev
0.1	8.0 ±	0.4	2.1 ±	0.5	6.0 ±	2.7	3.4 ±	1.4	17.8 ±	2.2	3.4 ±	1.4	37.3 ±	4.5	3.8 ±	0.1	5.0 ±	0.5
0.1	47.9 ±	0.8	0.0 ±	0.0	65.7 ±	8.6	52.9 ±	0.6	76.0 ±	2.0	52.9 ±	0.6	159.2 ±	3.9	43.8 ±	2.0	4.2 ±	0.7
0.2	355.5 ±	28.6	30.0 ±	42.4	216.3 ±	23.1	163.8 ±	25.0	173.4 ±	55.9	178.2 ±	3.1	496.0 ±	5.7	137.9 ±	7.3	3.9 ±	0.2
0.2	269.1 ±	14.9	175.3 ±	14.2	108.6 ±	1.7	309.5 ±	10.0	286.2 ±	25.9	309.5 ±	2.6	600.2 ±	44.0	261.0 ±	16.1	0.9 ±	0.1
0.3	457.9 ±	11.9	157.5 ±	51.1	351.6 ±	78.7	590.1 ±	5.9	478.1 ±	38.5	590.1 ±	5.9	1012.7 ±	80.1	496.0 ±	20.0	1.4 ±	0.1

	**NeoHookean**		**Mooney-Rivlin**		**Rubenstien-Panyuko**		**LM**		**Ogden***		**Swelling (v/v)**	
	R^2		R^2		R^2		R^2		R^2		Q	
Wt%.	Avg	± StDev	Avg	± StDev	Avg	± StDev	Avg	± StDev	Avg	± StDev	Avg	± StDev
0.1	0.01 ±	0.00	0.55 ±	0.07	0.27 ±	0.06	0.22 ±	0.03	0.96 ±	0.04	17.0 ±	0.0
0.1	0.01 ±	0.01	0.67 ±	0.06	0.65 ±	0.16	0.58 ±	0.10	1.00 ±	0.00	12.1 ±	0.0
0.2	-0.03 ±	0.01	0.69 ±	0.09	0.80 ±	0.08	0.80 ±	0.09	0.98 ±	0.02	8.4 ±	0.2
0.2	0.09 ±	0.02	0.81 ±	0.05	0.93 ±	0.02	0.92 ±	0.02	0.99 ±	0.00	6.0 ±	0.1
0.3	0.04 ±	0.34	0.24 ±	0.05	0.93 ±	0.01	0.95 ±	0.01	0.99 ±	0.00	4.7 ±	0.0

Table S4. - PEGDA (4000 Da) Modeling parameters

PEGDA (4000 Da) modeling parameters

	**NeoHookean**		**Mooney-Rivlin**				**Rubenstien-Panyukov**				**Localization Model**				**Ogden***			
	G		$2C_1$		$2C_2$		G_c		G_e		G_c		G_e		G		α	
Wt%.	Avg	± StDev	Avg	± StDev	Avg	± StDev	Avg	± StDev	Avg	± StDev	Avg	± StDev	Avg	± StDev	Avg	± StDev	Avg	± StDev
0.1	20.3 ±	3.4	16.7 ±	2.0	4.6 ±	6.7	9.2 ±	0.6	37.9 ±	3.0	9.2 ±	0.6	79.9 ±	6.6	11.7 ±	1.1	0.2 ±	0.2
0.1	62.2 ±	2.4	18.1 ±	0.1	62.6 ±	4.7	37.1 ±	2.6	106.7 ±	4.6	37.1 ±	2.6	225.1 ±	9.8	41.8 ±	2.2	3.0 ±	0.1
0.1	95.7 ±	5.9	99.9 ±	46.3	28.6 ±	31.4	63.7 ±	2.8	145.7 ±	10.5	63.7 ±	2.8	305.8 ±	22.3	56.5 ±	1.3	0.4 ±	0.2
0.3	247.4 ±	9.5	91.1 ±	42.8	177.6 ±	63.1	235.3 ±	17.4	361.7 ±	11.6	235.3 ±	17.4	767.3 ±	21.9	256.2 ±	12.3	3.1 ±	0.1

	**NeoHookean**		**Mooney-Rivlin**		**Rubenstien-Panyuko**		**LM**		**Ogden***		**Swelling (v/v)**	
	R^2		R^2		R^2		R^2		R^2		Q	
Wt%.	Avg	± StDev	Avg	± StDev	Avg	± StDev	Avg	± StDev	Avg	± StDev	Avg	± StDev
0.1	0.01 ±	0.01	0.02 ±	0.03	0.52 ±	0.08	0.49 ±	0.08	0.99 ±	0.01	16.2 ±	0.0
0.1	0.02 ±	0.06	0.51 ±	0.01	0.76 ±	0.08	0.71 ±	0.06	1.00 ±	0.00	13.6 ±	0.0
0.1	0.02 ±	0.05	0.14 ±	0.19	0.89 ±	0.02	0.88 ±	0.02	1.00 ±	0.01	12.8 ±	0.2
0.3	-0.01 ±	0.05	0.47 ±	0.00	0.87 ±	0.03	0.86 ±	0.01	1.00 ±	0.00	7.0 ±	0.0

Table S5. Overlap concentration of PEGDA

**Overlap concentration of PEGDA**	
MW (Da)	c* (wt%)
575	20
700	18
2000	12
4000	9
